# Photosulfoxidation Catalysis as the Driving Principle for Deazaoxaflavin Photoredox Catalyst Formation

**DOI:** 10.1021/acs.joc.5c00185

**Published:** 2025-09-25

**Authors:** Karolína Křížová, Rimeh Ismail, Tung Anh Nguyen, Marek Bříza, Anna O. Geleverya, Chiara Ciotta, Valentino L. P. Guerra, Petr Kovaříček

**Affiliations:** Department of Organic Chemistry, 430769 University of Chemistry and Technology Prague, Technická 5, 166 28 Prague 6, Czechia

## Abstract

Catalysts are essential for sustainability because they decrease energy and resource consumption in the production of high value-added products. The design of a novel catalyst is a challenging and expensive target, and a simplified methodology for catalyst development can trigger burgeoning progress in both academic and applied research. Here, we demonstrate a reaction network that autonomously yields the photoredox catalyst for the transformation of the provided substrate under applied catalytic conditions. The system stems from the reversible condensation pathway leading to deazaoxaflavins, 2*H*-chromeno­[2,3-*d*]­pyrimidine synthetic analogs of flavins, with which they share photoorganocatalytic activity. We report on the photocatalytic activity of deazaoxaflavins and their covalent dynamic behavior. The reversibility principle allows for the exchange of one of the deazaoxaflavin constituents for a different moiety, thus leading to adaptability of the catalyst. We argue that the observed phenomenon is of thermodynamic origin and thus can be applied to other photo/organocatalytic reactions in which the combination of a suitable substrate and conditions is the governing principle for catalyst formation.

## Graphical abstract

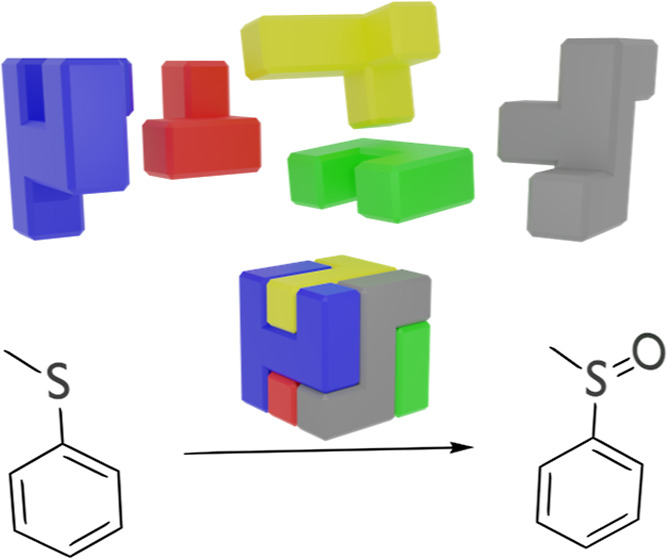

## Introduction

2*H*-chromeno­[2,3-*d*]­pyrimidines are referred to as deazaoxaflavins (DAOFs) due to their structural analogy with flavins, known cofactors of enzymes and artificial redox catalysts.^1–7^ They are formed by a sequence of reversible condensation reactions from derivatives of barbituric acid and salicylaldehyde.^8,9^ Despite the structural resemblance of DAOFs to flavins, their catalytic properties are unexplored. This inspired us to benchmark the catalytic properties of DAOFs vs flavins in typical redox catalytic assays—thioether oxidation to sulfoxide^10–13^ and haloarene dehalogenative reduction.^14^

The substitution for carbon in position 5 on the isoalloxazine skeleton creates a Michael acceptor arrangement, which readily reacts with nucleophiles, including C-nucleophiles such as barbituric acid.^15^ However, as a sequence of fully reversible reactions, the retro-Michael step has equal probability to yield products of elimination of both the original and the new moiety added. This opens the pathway for structural adaptation of DAOF catalysts to particular conditions applied.^16^

Engagement of the DAOF molecule in a catalytic cycle is, although transient, removal of a reversible cascade product and thus shifts the equilibrium in favor of DAOF catalyst formation.^17^ Here, we describe reversibly formed DAOF catalysts that autonomously assemble from their components. Their formation is governed thermodynamically by employment in a catalytic cycle; therefore, the catalytic function is the driving principle for catalyst formation (Figure 1).

**Figure 1:**
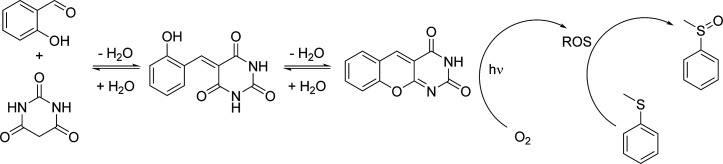
Scheme of the sequence of reversible reactions leading to the formation of the DAOF catalyst. When the DAOF product is employed in the catalytic cycle, the reversible sequence acts according to Le Chatelier’s principle and yields more of the catalyst.

An initial version of this work was deposited in ChemRxiv on March 15th, 2024, ref 18.

## Results and Discussion

The condensation of barbituric acid and salicylaldehyde derivatives yields DAOFs in two steps (Figure 2). First, the Knoevenagel reaction is performed in water at ambient temperature. Very mild conditions in this step are crucial because elevated temperature or the presence of a base or acid unequivocally leads to Michael addition of a second barbituric moiety to intermediate **a**. In the second step, pyran ring closure is promoted by acetic anhydride in acetic acid at 90 °C in 55–92% yield over the two steps. Derivatives with electron-donating alkoxy/hydroxy groups and electron-withdrawing nitro groups in various positions were prepared to estimate the electronic effect on the catalytic performance and to modulate solubility in organic solvents. Our results are benchmarked against tetraacetyl riboflavin (TARF), which is the most studied and common reference.

**Figure 2:**
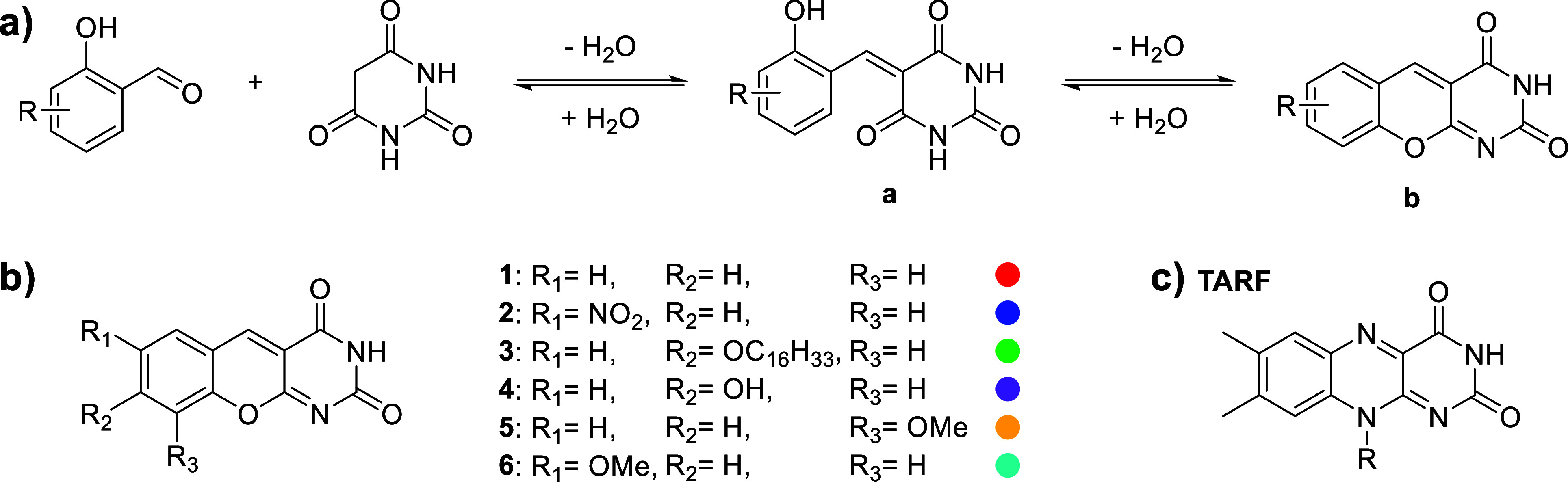
(a) Scheme of DAOF synthesis via two consecutive reversible condensations. While the Knoevenagel condensation to **a** proceeds in water at ambient temperature, the dehydrative cyclization to **b** in the second step requires heating to 90 °C in an AcOH/Ac_2_O mixture to yield preparative amounts of the product. (b) Structures of prepared DAOF derivatives **1**–**6**. The combination of letter and number indices indicates the condensation step and substitution, with color coding used in the following figures. (c) Structure of the molecule used as the reference, 2′,3′,4′,5′-tetraacetylriboflavin (TARF).

The photocatalytic performance of the prepared DAOF series was tested on two benchmark redox reactions established in flavin photocatalysis.^4^ The oxidative ability was tested by aerobic photosulfoxidation of thioanisole, while the reduction potential was evaluated by an aromatic dehalogenative assay. The progress of the photocatalytic reactions was monitored by HPLC. The oxidation of thioanisole to methyl phenyl sulfoxide was performed in an 85:15 acetonitrile—water mixture at a substrate concentration of 10 mM and 2 mol % catalyst loading. The solvent was saturated with O_2_ and kept under an oxygen atmosphere. Irradiation was performed with a 400 nm diode with an optical output power of 70 mW at 40 °C with stirring.

The nonsubstituted **1b**-catalyzed conversion of thioanisole to sulfoxide reached 70% in 30 min (Figure 3a), similarly to **6b** with a methoxy substituent as R_1_. **3b** and **5b** showed lower conversion, not exceeding 40% in 30 min. Electron-poor **2b** was the least efficient, giving only ca 10% conversion at the same time interval. Interestingly, **4b** with the hydroxy group in position R_1_ showed complete conversion of the starting material to sulfoxide within 5 min, which even exceeds the photocatalytic activity of TARF (Figure S8). Negative controls (see Supporting Information Section 5.1.1 and Figure S9) showed no conversion without catalyst, light, or oxygen present in the mixture. Under the reaction conditions, there is no evidence of catalyst degradation; the catalyst remains stable and effective throughout the experiment, with no significant loss of activity.

**Figure 3:**
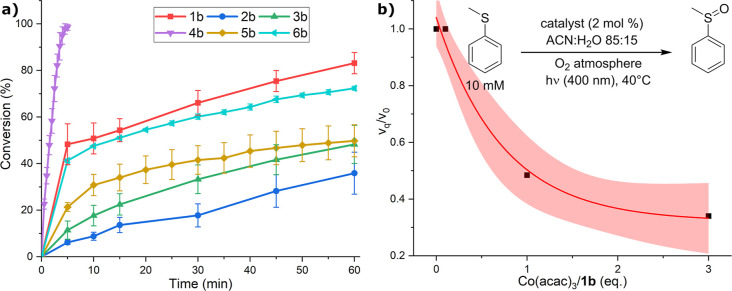
(a) Photocatalytic oxidation of thioanisole to sulfoxide by DAOF derivatives. The curves and their error bars are calculated as the average and standard deviation, respectively, of three independent runs. The TARF reference is in Figure S8. (b) Effect of the singlet oxygen quencher [Co­(acac)_3_] addition on the relative rate of photosulfoxidation (black squares are experimental data, and the red line is the fitting with the embedded confidence interval set as 68%). In the inset, a scheme of the photooxidative reaction tested, with the detailed conditions.

For DAOFs, 400 nm irradiation is at the very edge of the most bathochromic absorbance band, while for TARF, it exceeds the HOMO–LUMO gap energy by ca 0.5 eV (see Supporting Information Section 3). Importantly, catalysis by TARF requires water in the solvent to proceed, but DAOFs also catalyze photosulfoxidation in pure organic solvents with 70% conversion within 2 h (see Supporting Information Figure S7). DAOFs are thus better performing and have a broader applicability range than widely employed TARF.

While the effect of electron-donating/withdrawing substituents on the DAOF skeleton is ambiguous in aqueous environments (Figure 3), a significantly faster reaction was observed in pure acetonitrile for the **3b** derivative (Supporting Information Figure S7). We determined the HOMO–LUMO energies of DAOFs from cyclic voltammetry and UV–vis absorbance onset to understand the reactivity and referenced them to TARF. DAOFs feature deeper HOMO levels by up to 0.9 eV and a wider gap by up to 0.5 eV than TARF. The photosulfoxidation quantum yield (ϕ_ox_) ranges from 0.03 for **2b** to 0.65 for **4b** (Supporting Information Table S1). ϕ_ox_ correlates with the determined E_HOMO_ energy, with **4b** featuring the lowest HOMO (−6.77 eV). From this correlation we deduce that the electron transfer (ET) catalytic cycle operates under an oxidative quenching regime.^19^ For **2b** (−6.18 eV) and **3b** (−5.91 eV) the trend is ambiguous, which we attribute to the solubility of the catalyst in the solvent of choice, as evidenced by the high performance of **3b** featuring a long aliphatic chain in less polar environments. However, it has been shown with TARF that ET and singlet oxygen (^1^O_2_) mechanisms can compete in the photosulfoxidation process.^4^ We have conducted photosulfoxidation experiments with **1b** in increasing amounts of Co­(acac)_3_ as a singlet oxygen quencher.^4^ The initial reaction rates were determined and plotted as a function of equivalents of Co­(acac)_3_ with respect to **1b** (Figure 3b). Significant deceleration of the reaction was observed with 1 and 3 equiv; however, the nonzero rate limit indeed indicates that both ET and ^1^O_2_ mechanisms are involved in the reaction approximately at 1:3 ratio, respectively.

The reductive catalytic assay followed the conversion of 4-bromoanisole to anisole. The reaction is overly sensitive to residual oxygen, and thus, pure and freeze–pump–thaw degassed acetonitrile solutions were used. *N,N*-Diisopropylethylamine (DIPEA, 2 equiv) as the sacrificial electron donor and cesium carbonate (1 equiv) as a base were added as well as 8 mol % catalyst, and the mixture was irradiated with a 400 nm diode at 70 mW optical output power. Despite higher catalyst loading compared to the oxidative assay, the reduction is considerably slower, and a conversion of 7% was only reached after 24 h of irradiation (see Supporting Information Figure S11).

The synthesis of DAOF commences by principally reversible Knoevenagel condensation of barbituric acid with salicylaldehyde; however, the resulting α,β-unsaturated ketone **1a** is a good Michael acceptor for the addition of the second barbiturate. In the following retro-Michael step, there is an equal probability for elimination of either of the barbiturates, thus leading to an equilibrium mixture consisting of both Michael adducts and retro-Michael products (Figure 4). We prepared a DMSO-*d*_6_ solution of **1a** (20 mM), added 1 eq. of *N*,*N*′-dimethylbarbituric acid, and followed the evolution by NMR (Figure S14). Equilibrium was established over a period of 48 h at ambient temperature and consisted of 37% **1a**, 35% of its *N,N*′-dimethylated analogue, and three adducts of aldehyde with two barbiturates (nonmethylated 10%, dimethylated 7%, and tetramethylated 11%). **1b** also features the Michael acceptor, and indeed, the addition of a second barbituric moiety proceeds in DMSO-*d*_6_ with full conversion within a minute, so equilibrium is reached before the first NMR spectrum can be acquired. This was found to be common for all species in this study, and NMR spectra recorded in strong solvent (DMSO) always show reequilibration of **a** or **b** and their corresponding Michael adducts as constituents of the reaction network.^16,17^

**Figure 4:**
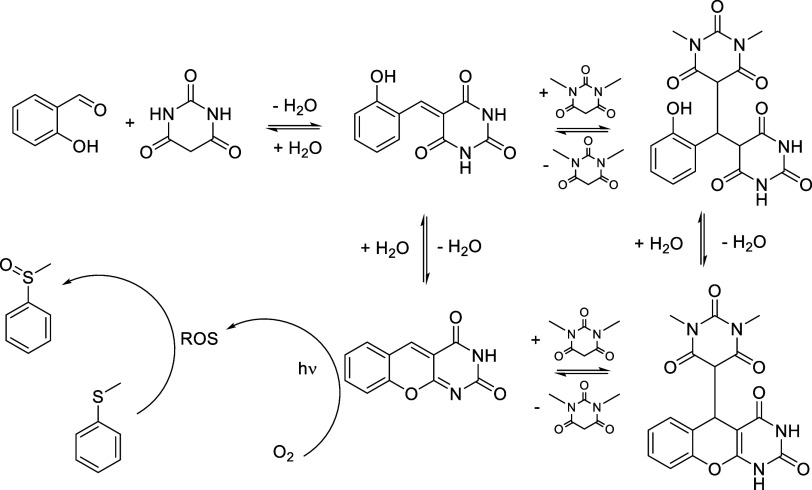
Scheme of the dynamic covalent network of DAOF derivatives. Three reversible processes (two condensations and one conjugated addition) are operative. Involvement of the resulting DAOF **1b** in the catalytic cycle drives the equilibria toward its formation, as described below.

The covalent dynamicity of DAOFs inspired us to test the hypothesis of whether the catalytic function of **1b** can serve as the driving principle for its formation. We therefore replaced **1b** in the photocatalytic experiment with its uncondensed precursor **1a** and repeated the sulfoxidation assay under otherwise identical conditions. Monitoring of the photocatalysis (Figure 5, red curve) showed a sigmoidal curve of thioanisole conversion, indicative of an induction of the catalyst in the early stages of the reaction. This suggests that **1a** condenses to **1b** during the catalytic experiment and that **1b** is then responsible for the observed formation of the product. While the preparative scale reaction converting **1a** to **1b** required heating (90 °C) and dehydrating solvent (AcOH/Ac_2_O), here, the conversion is achieved in aqueous acetonitrile at 40 °C upon irradiation. The photocatalytic sulfoxidation showed that 90% conversion was achieved in 180 min in the run with **1a**, while with **1b,** similar conversion was reached in 90 min.

**Figure 5:**
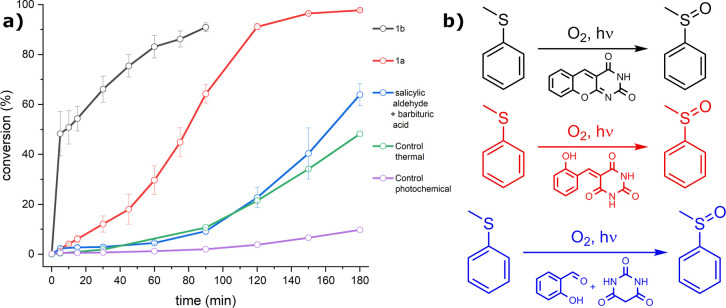
Monitored photocatalytic oxidation of thioanisole. The black trace shows the reaction using **1b** as the reference. The red trace corresponds to the reaction with **1a**. The blue trace reports on the reaction mixture containing salicylaldehyde, barbituric acid, and thioanisole. The sigmoidal shape of the red and blue curves indicates catalyst formation during the reaction. The green trace “Control thermal” involves 3 h preheating of the reaction mixture containing salicylaldehyde, barbituric acid, and thioanisole at 40 °C without irradiation before the catalytic run. The purple trace “Control photochemical” reports on the mixture of barbituric acid and salicylaldehyde preirradiated for 3 h without thioanisole, then thioanisole was added, and the photosulfoxidation was followed.

However, even the formation of **1a** is governed thermodynamically; thus, we have taken the concept further. We have mixed **1a** precursors, i.e., salicylaldehyde and barbituric acid, thioanisole was added, the solution was purged with O_2_ for 1 min, and photocatalytic sulfoxidation was monitored by HPLC (Figure 5, blue curve). We again observed gradually increasing conversion following a sigmoid curve, yet in this case with a prolonged initial latency period, again indicative of catalyst induction in the reaction. Essential negative control experiments without the catalyst or oxygen showed again no photosulfoxidation conversion at all.

The results from photocatalysis kinetics show that a reaction network (Figure 1) is established in the mixture containing the photocatalyst precursors, substrate (thioanisole), and reagent (oxygen). Irradiation with light drives the system out of equilibrium to form a photoactive species, which enters the catalytic cycle. We have confirmed the formation of both intermediate **1a** and photocatalyst **1b** in such a mixture under photocatalytic conditions by HRMS (Supporting Information Section 5.3).

One could object that some amounts of **1a** and **1b** form spontaneously in aqueous acetonitrile at 40 °C, i.e., the conditions under which the photocatalysis runs. To verify this objection, we performed a series of control experiments. First, we have mixed salicylaldehyde and barbituric acid at a 0.2 mM concentration level used in the catalysis in a CD_3_CN/D_2_O 85:15 solvent mixture. After heating the mixture at 40 °C for 3 h, the NMR spectrum did not show any trace of **1b** or **1a**. Formation of these products was only observable by NMR after the reaction time was prolonged to 24 h (Supporting Information, Figure S15), when also complete photosulfoxidation conversion was reached.

Second, we prepared a mixture of salicylaldehyde and barbituric acid (both 0.2 mM) and thioanisole (10 mM) in analogy to 2% catalyst loading during the photosulfoxidation runs of **1–6b** (Figure 3). This mixture was purged with oxygen and then heated at 40 °C for 3 h without irradiation, which did not lead to any sulfoxide formation. However, when we exposed this mixture to 400 nm light and sampled it over 180 min, a sigmoidal conversion curve was obtained (Figure 5, green trace “control thermal”). Moreover, this curve matched perfectly the one obtained without the thermal pretreatment. This confirms that neither **1b** nor **1a** is formed during the thermal pretreatment phase under the conditions applied. Their catalytically significant formation is only triggered when the catalytic cycle can operate, i.e., when catalyst precursors, substrate, oxygen, and light are administered simultaneously.

Finally, we have probed the hypothesis that **1a** and **1b** form photochemically from their precursors by excitation with 400 nm light, although the absorbance edge of these compounds is far below this wavelength. We have thus again mixed salicylaldehyde and barbituric acid (both 0.2 mM), purged the mixture with oxygen, and irradiated it with 400 nm light (70 mW) at 40 °C for 3 h. Then, we injected thioanisole (to reach 10 mM concentration), continued the irradiation for 3 h, and followed the photosulfoxidation in time. Slow conversion of thioanisole to sulfoxide was observed (Figure 5, purple curve “control photochemical”), which excludes the possibility of preformation of either **1a** or **1b** photochemically without the presence of the photocatalysis substrate thioanisole.

Thioanisole photooxidation to sulfoxide by molecular oxygen catalyzed by DAOFs, similarly to flavins, proceeds by both ET and ^1^O_2_ mechanisms, either a Type I or Type II ^1^O_2_ mechanism.^1,4^ An important side product of these reactions is the formation of hydrogen peroxide, which is a rather good nucleophile. The presence of the Michael acceptor motif in the structure of DAOF allows for the reversible Michael addition of hydroperoxide in analogy to Figure 4.

We have added H_2_O_2_ to **1b** and recorded UV–vis and NMR spectra. In UV–vis, a gradual decrease of the most bathochromically shifted absorbance band of DAOF was observed when aliquots of H_2_O_2_ were added (Figure S16). A decrease in this band suggests disruption of a conjugated system, which is consistent with the Michael addition process (Figure 4). NMR spectra of **1b** after addition of hydrogen peroxide (Figure S 17) show the shift of the central C–H proton from 8.95 to 5.71 ppm, indicating a change of the character from a phenylvinylene-like proton to a benzylic proton. This is accompanied by the appearance of a new N–H signal with a significantly broader line shape than the second N–H proton, indicative of a dynamic exchange other than basic proton exchange with the solvent.

These findings allow us to draw the following scenario: a mixture of salicylaldehyde and barbituric acid (or their derivatives) in aqueous solution establishes a reaction network with their condensation products **a** and **b**, although the equilibrium is almost completely shifted toward the starting materials. Yet, if conditions applied, i.e., substrate, reagent, and irradiation wavelength, match the properties of a component of this reaction network (**1b** in this case), the photocatalytic process will be triggered. After the catalytic cycle is performed, the restored catalyst reversibly reacts with a side product of the photocatalytic cycle (hydrogen peroxide in this case). This effectively removes the catalyst from the equilibrium reaction network. In response, the network reacts according to Le Chatelier’s principle by increasing the formation of the catalyst. As a result, induction of photocatalysis is achieved. We thus conclude that catalyst **1b** assembles under thermodynamic control from a reaction network during the photocatalytic process and that its formation is driven by its catalytic function.

## Conclusions

Our study shows the photocatalytic performance, dynamic covalent behavior and thermodynamically driven autonomous catalyst formation of deazaoxaflavin derivatives. DAOFs show activity in photosulfoxidations higher than or comparable to riboflavin, activity in dehalogenative reductions, and tolerance to a broader range of solvents. Unlike riboflavin, DAOFs exhibit covalent dynamicity, which imbues them with a responsiveness and adaptability. The constitutional adaptation^16,17,20,21^ was demonstrated in a scenario in which two simple compounds, salicylaldehyde and barbituric acid, spontaneously form a catalytic system due to the presence of a suitable substrate and feasible conditions. The catalytic function of the product is the driving principle for its formation. The photocatalytic oxidation product is a chiral sulfoxide; yet, it is produced as a racemate in our setup. Given the simplicity of the starting materials in the presented case, it can be hypothesized that similar conditions could have been achieved even prebiotically.^22–37^ We focus on expanding the results to other photocatalytic reactions, heterogeneous conditions, and enantioselective catalysis.

## Supplementary Material



## Data Availability

The data underlying this study are available in the published article, in its Supporting Information, and openly available in Zenodo at DOI 10.5281/zenodo.15781643.

## Notes

Materials used, synthetic protocols with NMR and HRMS data, determination of HOMO and LUMO energies from cyclic voltammograms and absorbance onset, UV–vis absorption and emission spectroscopy, photocatalysis details, and negative controls (PDF)
