# Supplementary material for: Photosulfoxidation Catalysis as the Driving Principle for Deazaoxaflavin Photoredox Catalyst Formation
Source: J Org Chem. 2025 Sep 25;90(40):14039–44. doi: 10.1021/acs.joc.5c00185 (PMC12519482; doi:10.1021/acs.joc.5c00185)
Supplement: Supplementary file 1 [file jo5c00185_si_001.pdf]

Photosulfoxidation catalysis as the driving principle for the deazaoxaflavin photoredox catalyst formation

Supporting information file

*Karolína Křížová, Rimeh Ismail, Tung Anh Nguyen, Marek Bříza, Anna O. Geleverya, Chiara Ciotta, Valentino L. P. Guerra, Petr Kovaříček**

University of Chemistry and Technology Prague, Department of Organic Chemistry, Technická 5, 166 28, Prague 6, Czechia; petr.kovaricek@vscht.cz, +420 220 444 165

1 Materials S2

2 Synthesis S2

2.1 Synthesis of deazaoxaflavins (DAOFs) S2

2.1.1 Synthesis of 5-(2-hydroxybenzylidene)pyrimidine-2,4,6-trione (**1a**) S3

2.1.2 Synthesis of 2H-chromeno[2,3-d]pyrimidine-2,4-dione (**1b**) S4

2.1.3 Synthesis of 5-(2-hydroxy-5-nitrobenzylidene)pyrimidine-2,4,6-trione (**2a**) S6

2.1.4 Synthesis of 7-nitro-2H-chromeno[2,3-d]pyrimidine-2,4-dione (**2b**) S8

2.1.5 Synthesis of 4-(hexadecyloxy)-2-hydroxybenzaldehyde S10

2.1.6 Synthesis of 5-(4-hexadecyloxy-2-hydroxybenzylidene)pyrimidine-2,4,6-trione (**3a**) S12

2.1.7 Synthesis of 8-hexadecyloxy-2H-chromeno[2,3-d]pyrimidine-2,4-dione (**3b**) S14

2.1.8 Synthesis of 5-(2,4-dihydroxybenzylidene)pyrimidine-2,4,6-trione (**4a**) S16

2.1.9 Synthesis of 8-hydroxy-2H-chromeno[2,3-d]pyrimidine-2,4-dione (**4b**) S18

2.1.10 Synthesis of 5-(2-hydroxy-3-methoxybenzylidene)pyrimidine-2,4,6-trione (**5a**) S20

2.1.11 Synthesis of 9-methoxy-2H-chromeno[2,3-d]pyrimidine-2,4-dione (**5b**) S22

2.1.12 Synthesis of 5-(2-hydroxy-5-methoxybenzylidene)pyrimidine-2,4,6-trione (**6a**) S24

2.1.13 Synthesis of 7-methoxy-2H-chromeno[2,3-d]pyrimidine-2,4-dione (**6b**) S26

3 Determination of HOMO and LUMO energies S29

4 Spectroscopy S33

5 Photocatalysis S35

5.1 Catalytic oxidations S35

5.1.1 Negative controls S38

5.2 Catalytic reductions S38

5.2.1 Negative controls S39

5.3 Autonomous catalyst formation under catalytic conditions S40

1 Materials

Solvents and starting materials were purchased from Merck, Roth, Penta and used as received.

Deoxygenation was performed as follows: 1.) reaction mixture in a closed two-neck Schlenk tube is frozen in liquid nitrogen and then put under vacuum, 2.) the frozen liquid is thawed and when fully liquid put under inert gas atmosphere, 3.) the process is repeated three times, 4.) In the last cycle, inert gas is introduced during thawing of the solution.

HPLC chromatograms were recorded on a Knauer system with an autosampler and an Astra C18-HE 100x4.6 mm, 3 µm column with isocratic elution (H_2O:MeOH 60:40) and recorded with Clarity software.

Elution was performed with isocratic MeOH:water (40:60), and sulfoxidations were monitored at 232 nm. For reductions, isocratic ACN:water (60:40) and monitoring at 274 nm were used. Thirty microliters of the photocatalytic reaction mixture was diluted in 1.5 mL of pure acetonitrile, and 5 µL of this diluted sample was injected onto an HPLC column.

NMR spectra were acquired on two spectrometers: ^{1}H NMR spectra were measured on Agilent 400 MHz, and JEOL JNM-ECZL400G at 400 MHz, ^{13}C NMR spectra were measured on a JEOL JNM-ECZL400G instrument at 101 MHz. Spectra were referenced to the residual solvent signal according to the literature,[26] and chemical shifts are given in ppm. The spectra were processed in MestreNova software.

Absorption spectra were recorded on an Agilent HP 8454 spectrometer in the range of 200-1000 nm at room temperature. Photoluminescence data (emission and excitation spectra) were recorded on a Varian Cary Eclipse at room temperature. All UV-vis and fluorescence measurements were performed in a quartz cuvette with a standard path of 1.00 cm in UV grade acetonitrile.

Cyclic voltammetry (CV) was performed using a PalmSens4 potentiostat with PSTrace software. Pt wire was used as the counter electrode, and a Pt mesh was used as the working electrode. Ag/AgCl (treated in 1 M HCl) was used as the pseudoreference electrode. All measurements were performed at room temperature in acetonitrile solution containing 0.08 M tetrabutylammonium hexafluorophosphate (Bu_4NPF_6).

2 Synthesis

2.1 Synthesis of deazaoxaflavins (DAOFs)

DAOFs are obtained in a two-step synthesis. First, a Knoevenagel condensation between a barbituric acid derivative and a salicylaldehyde derivative proceeds almost quantitatively in water at ambient temperature. In the second step, pyrane ring closure is performed in a mixture of acetic acid/acetic anhydride at elevated temperatures to obtain the desired DAOF **b** derivative. Derivatives **3** bearing hexadecyloxy chains are not sufficiently soluble/miscible with water, and the first condensation reaction was conducted in an acetonitrile:water mixture. Addition of acid or base catalyst or increased temperature always yielded the product of Michael addition to both the **a** intermediate and **b** product for

all derivatives in various ratios. Note: NMR spectra show trace amounts (<5 %) of 'impurities' – these were identified as products of covalently dynamic reequilibration discussed above. For example, **2b** reequilibrates in DMSO-d_6 faster than the time needed to record the ^{1}H spectrum, while the solubility of DAOFs in other NMR solvents is very limited. Reequilibration of species is natural in dynamic reaction networks, cannot be avoided and supports the presented claims.

*2.1.1 Synthesis of 5-(2-hydroxybenzylidene)pyrimidine-2,4,6-trione (**1a**)*

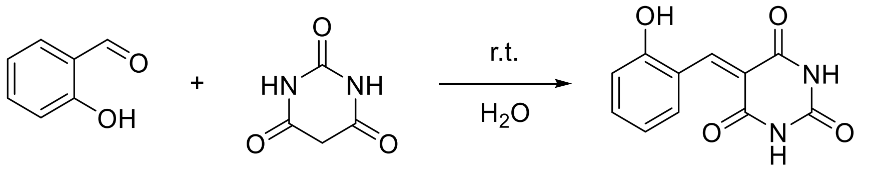

Barbituric acid (2.4 g, 18.7 mmol) was dispersed in 20 mL of distilled water, and salicylaldehyde (2.3 g, 18.7 mmol, 1 eq.) was added to the reaction mixture. This suspension was stirred for 30 minutes at r.t. A color change from yellow to deep orange was observed. The resulting suspension was filtered off, washed with water and ethanol, and dried under vacuum to yield 95% of product **1a** (4.1 g, 17.7 mmol). The analysis is in agreement with the literature reference.[27]

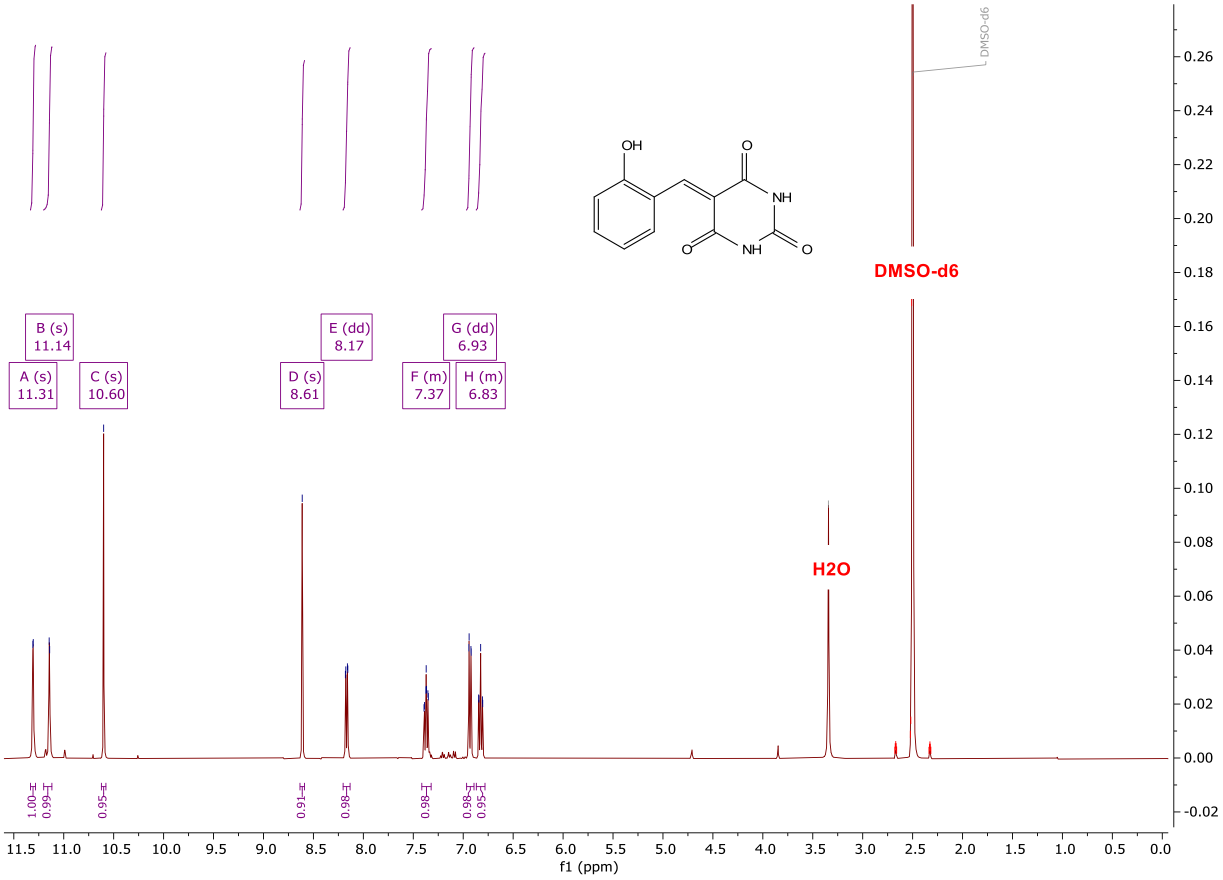

^{1}H NMR (400 MHz, DMSO-d_6) δ 11.31 (s, 1H), 11.14 (s, 1H), 10.60 (s, 1H), 8.61 (s, 1H), 8.17 (dd, *J* = 8.0, 1.7 Hz, 1H), 7.41 – 7.32 (m, 1H), 6.93 (dd, *J* = 8.4, 1.1 Hz, 1H), 6.87 – 6.78 (m, 1H).

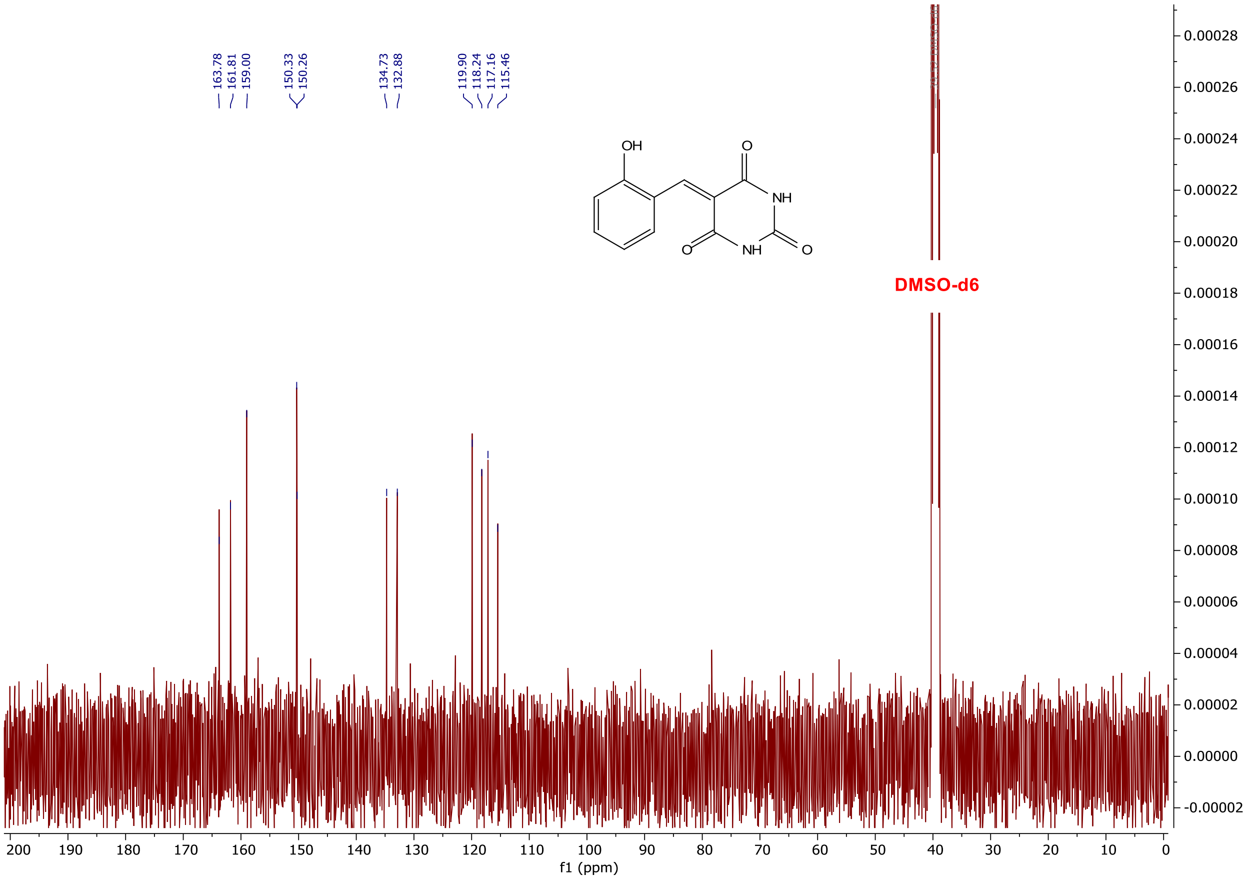

$^{13}C\{^{1}H\}$ NMR (101 MHz, DMSO-d_6) δ: 163.8, 161.8, 159.0, 150.3, 150.3, 134.7, 132.9, 119.9, 118.2, 117.2, 115.5.

HRMS (ESI-) m/z $[M - H]^-$: calculated for $C_{11}H_7N_2O_4$ 231.0411, found 231.0421

2.1.2 *Synthesis of 2H-chromeno[2,3-d]pyrimidine-2,4-dione (**1b**)*

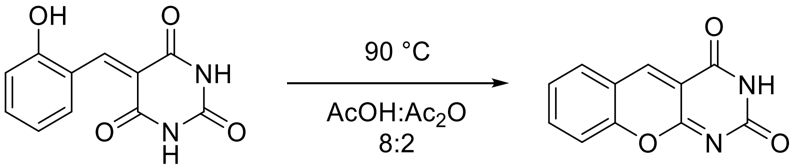

One gram (4.3 mmol) of compound **1a** was stirred with 10 mL of an acetic acid:acetic anhydride mixture (8:2) at 90°C for 2 hours on a hot plate with heating mantle. The color changed from orange to yellow. After cooling, the suspension was filtered. The filtered solid was washed with water and ethyl acetate to yield 93 % of the product **1b** (0.91 g, 4.3 mmol). The analysis is in agreement with the literature reference.[28]

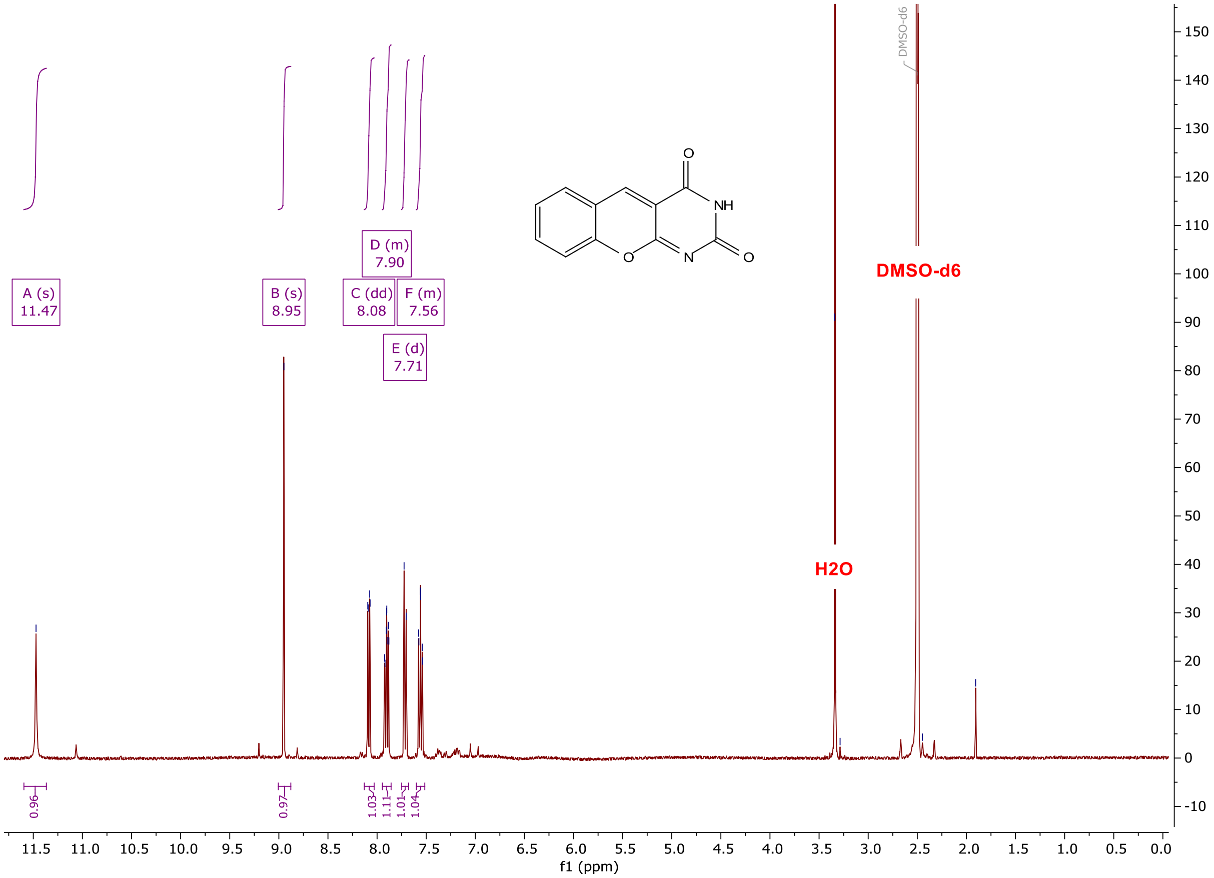

^{1}H NMR (400 MHz, DMSO-d_6) δ 11.47 (s, 1H), 8.95 (s, 1H), 8.08 (dd, *J* = 7.8, 1.6 Hz, 1H), 7.95 – 7.86 (m, 1H), 7.71 (d, *J* = 8.7 Hz, 1H), 7.60 – 7.51 (m, 1H).

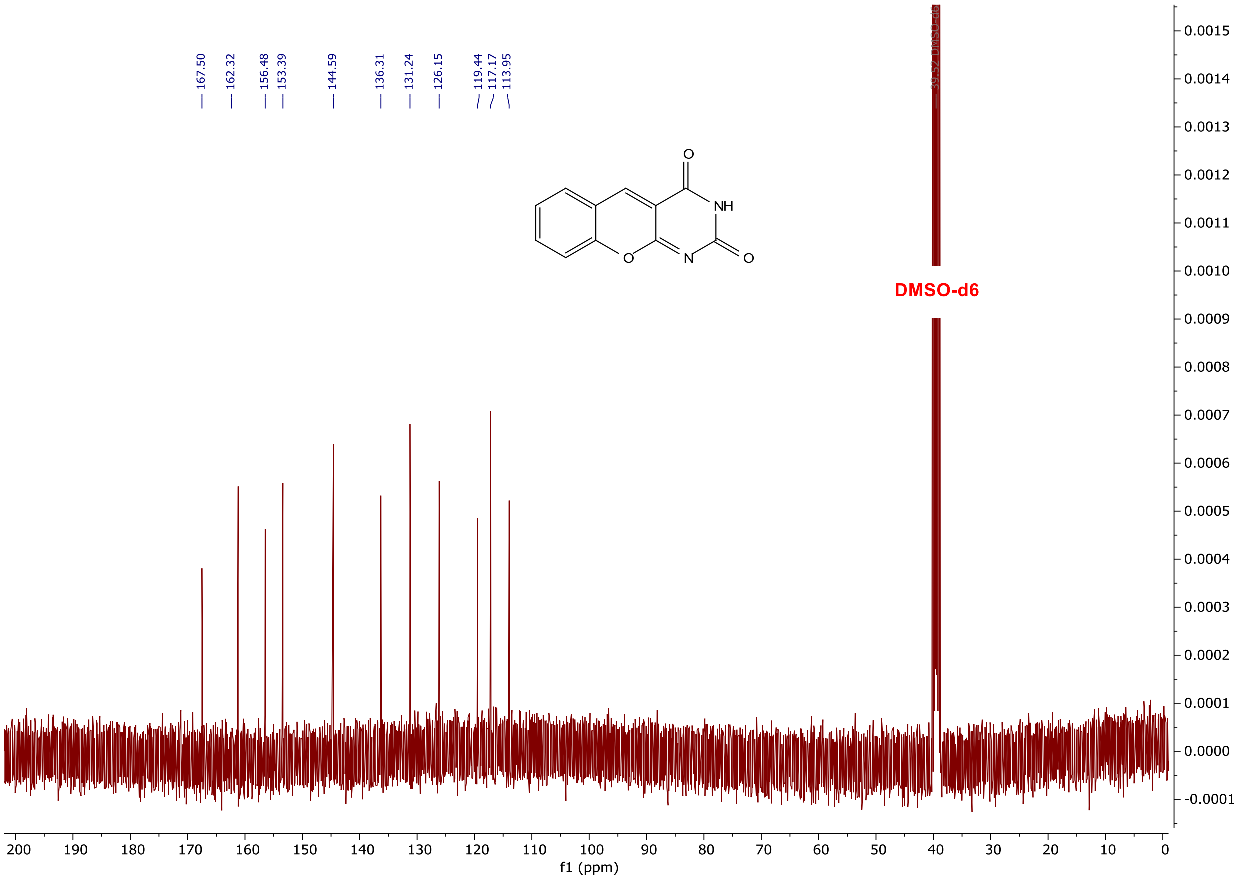

$^{13}C\{^1H\}$ NMR (101 MHz, DMSO-d_6) δ 167.5, 162.3, 156.5, 153.4, 144.6, 136.3, 131.2, 126.2, 119.4, 117.2, 114.0.

HRMS (ESI-) m/z $[M - H]^-$: calculated for $C_{11}H_5N_2O_3$ 213.0306, found 213.0315

*2.1.3 Synthesis of 5-(2-hydroxy-5-nitrobenzylidene)pyrimidine-2,4,6-trione (**2a**)*

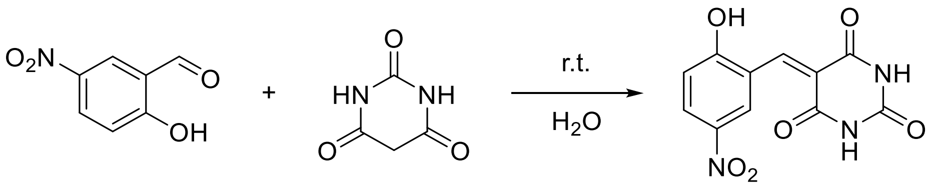

Barbituric acid (0.50 g, 3.9 mmol) was dispersed in 20 mL of water. Then, 2-hydroxy-5-nitrobenzaldehyde (0.66 g, 4 mmol) was added to the reaction mixture, and the suspension was stirred for 16 hours. A color change from slight yellow to orange occurs. Product **2a** was obtained as powder (60 %, 0.64 g, 2.34 mmol). The analysis is in agreement with the literature.[27]

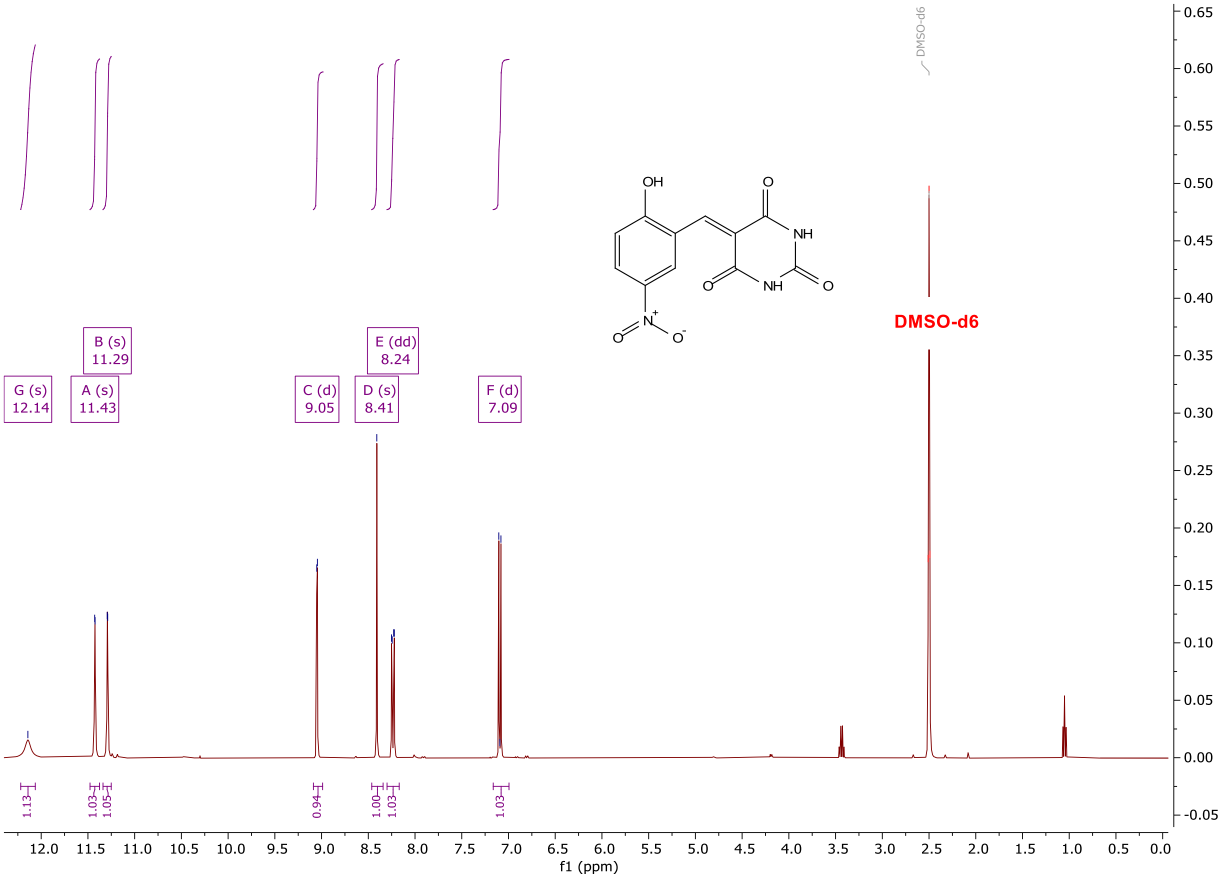

^{1}H NMR (400 MHz, DMSO-d_6) δ 12.14 (s, 1H), 11.43 (s, 1H), 11.29 (s, 1H), 9.05 (d, *J* = 2.8 Hz, 1H), 8.41 (s, 1H), 8.24 (dd, *J* = 9.1, 2.9 Hz, 1H), 7.09 (d, *J* = 9.2 Hz, 1H).

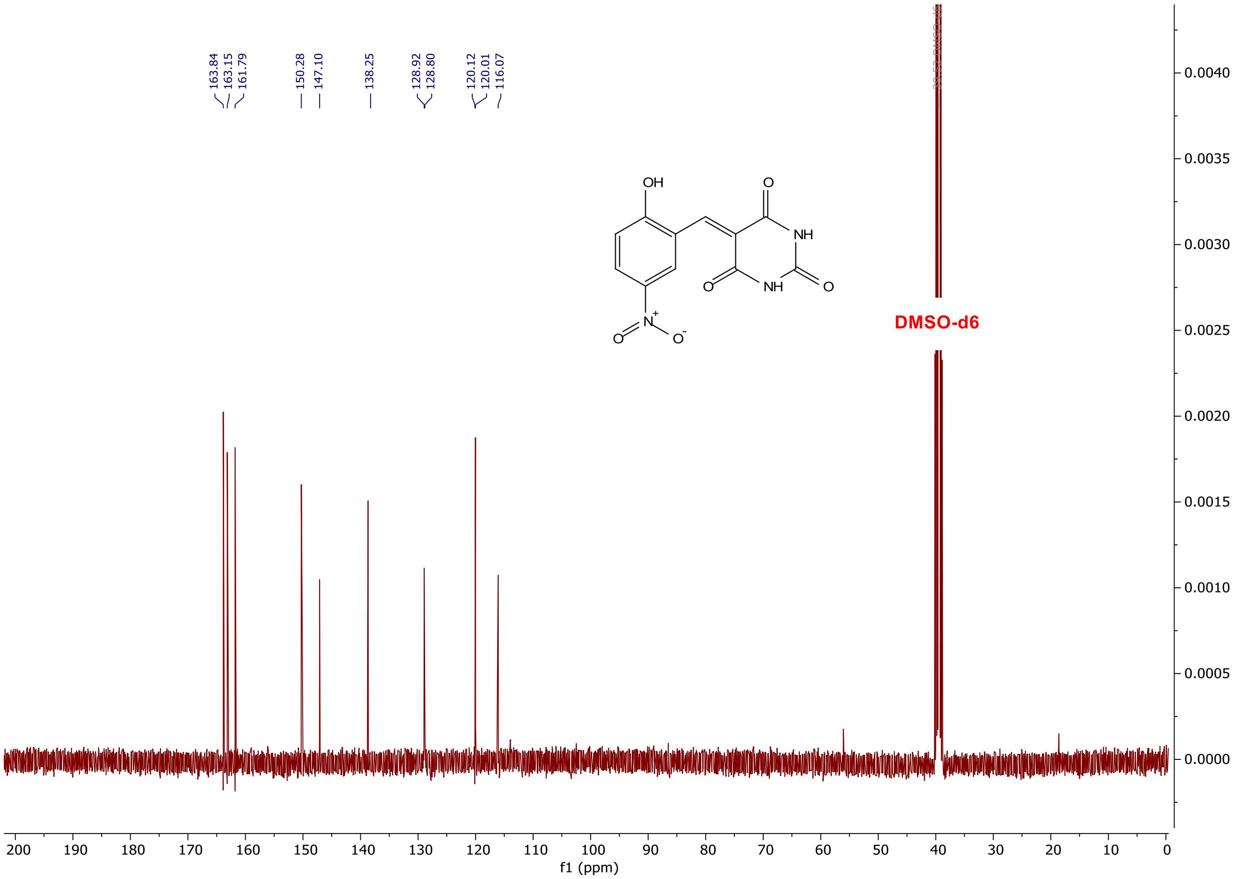

$^{13}C\{^1H\}$ NMR (101 MHz, DMSO-d_6) δ 163.8, 163.2, 161.8, 150.3, 147.1, 138.3, 128.9, 128.8, 120.1, 120.0, 116.1.

HRMS (ESI-) m/z $[M - H]^-$: calculated for $C_{11}H_6N_3O_6$ 276.0262, found 276.0275

*2.1.4 Synthesis of 7-nitro-2H-chromeno[2,3-d]pyrimidine-2,4-dione (**2b**)*

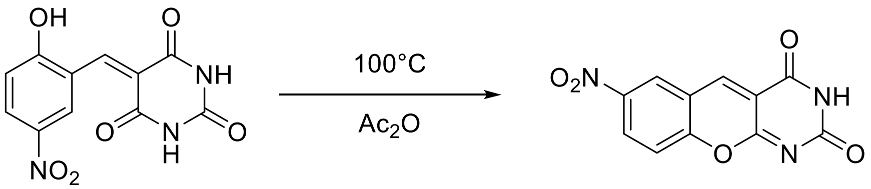

The cyclization of **2a** (1g, 3.6 mmol) was carried out in acetic anhydride at 100°C for 14 hours on a hot plate with heating mantle. The precipitate that formed was filtered, washed with ethyl acetate, and dried under vacuum. Product **2b** was obtained as a yellow powder (92 %, 0.85 g, 3.3 mmol).

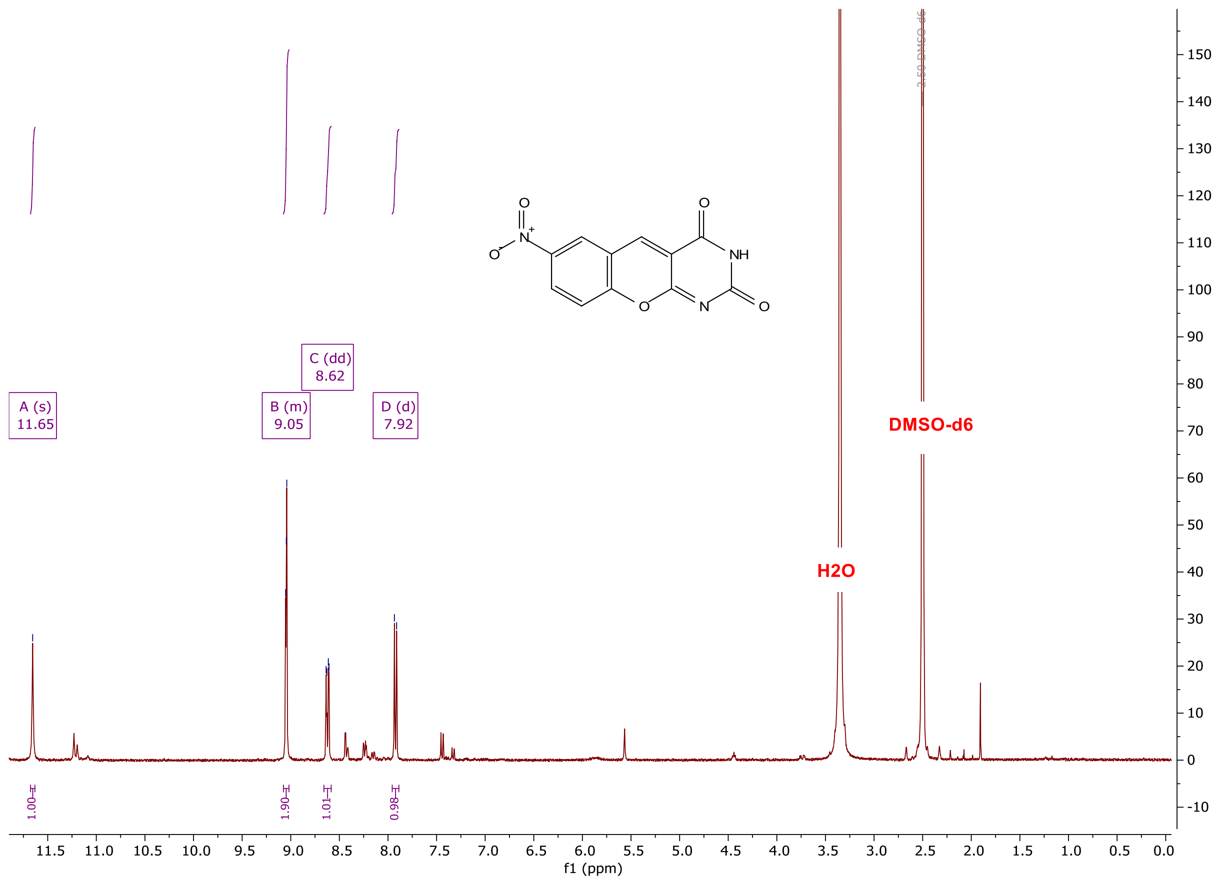

^{1}H NMR (400 MHz, DMSO-d_6) δ 11.65 (s, 1H), 9.08 – 9.02 (m, 2H), 8.62 (dd, *J* = 9.2, 2.8 Hz, 1H), 7.92 (d, *J* = 9.1 Hz, 1H).

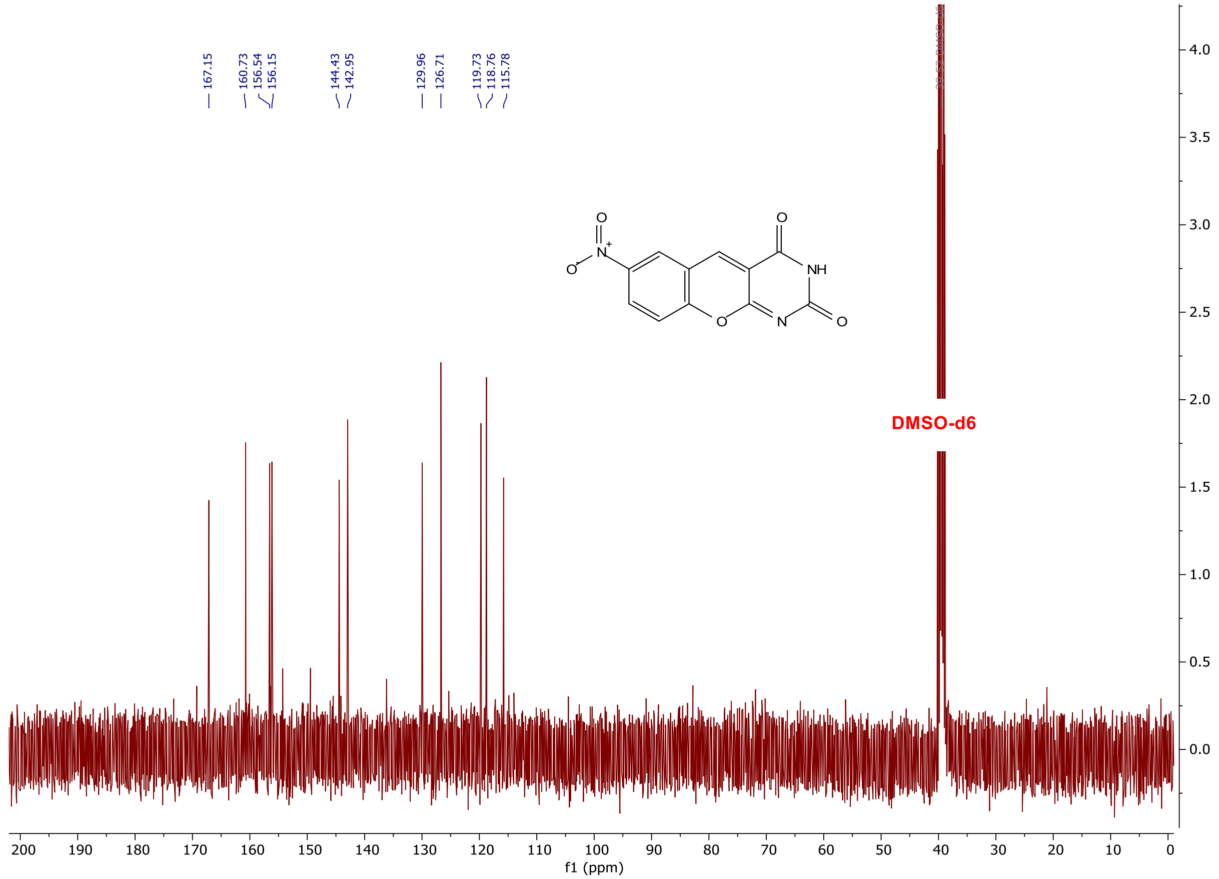

$^{13}C\{^{1}H\}$ NMR (101 MHz, DMSO-d_6) δ: 167.2, 160.7, 156.5, 156.2, 144.4, 143.0, 130.0, 126.7, 119.7, 118.8, 115.8.

HRMS (ESI-) m/z $[M - H]^-$: calculated for $C_{11}H_4N_3O_5$ 258.0156, found 258.0151

2.1.5 Synthesis of 4-(hexadecyloxy)-2-hydroxybenzaldehyde

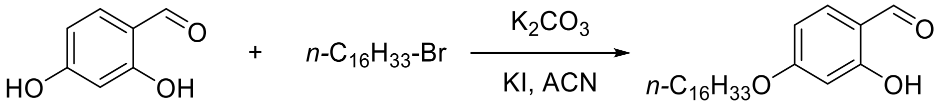

2,4-Dihydroxybenzaldehyde (1.00 g, 7.24 mmol), 1-bromohexadecane (2.21 g, 7.24 mmol), K_2CO_3 (1.00 g, 7.24 mmol) and a catalytic amount of KI (0.12 g, 0.72 mmol) were mixed in dry acetonitrile (250 mL), and the mixture was refluxed for 48 h on a hot plate with heating mantle. Then, it was filtered while hot to remove the insoluble solid. The warm solution was neutralized by adding diluted HCl and extracted twice with 100 mL of $CHCl_3$. The combined extracts were evaporated to obtain a yellow liquid. The product was further purified by column chromatography using silica gel (60-120 mesh) eluting with a gradient mixture of hexane and dichloromethane (v/v 4:1→1:1) followed by evaporation of the solvent. The product was obtained as a white solid. Yield = 69% (1.80 g, 4.96 mmol). The analysis is in agreement with the literature.[29,30]

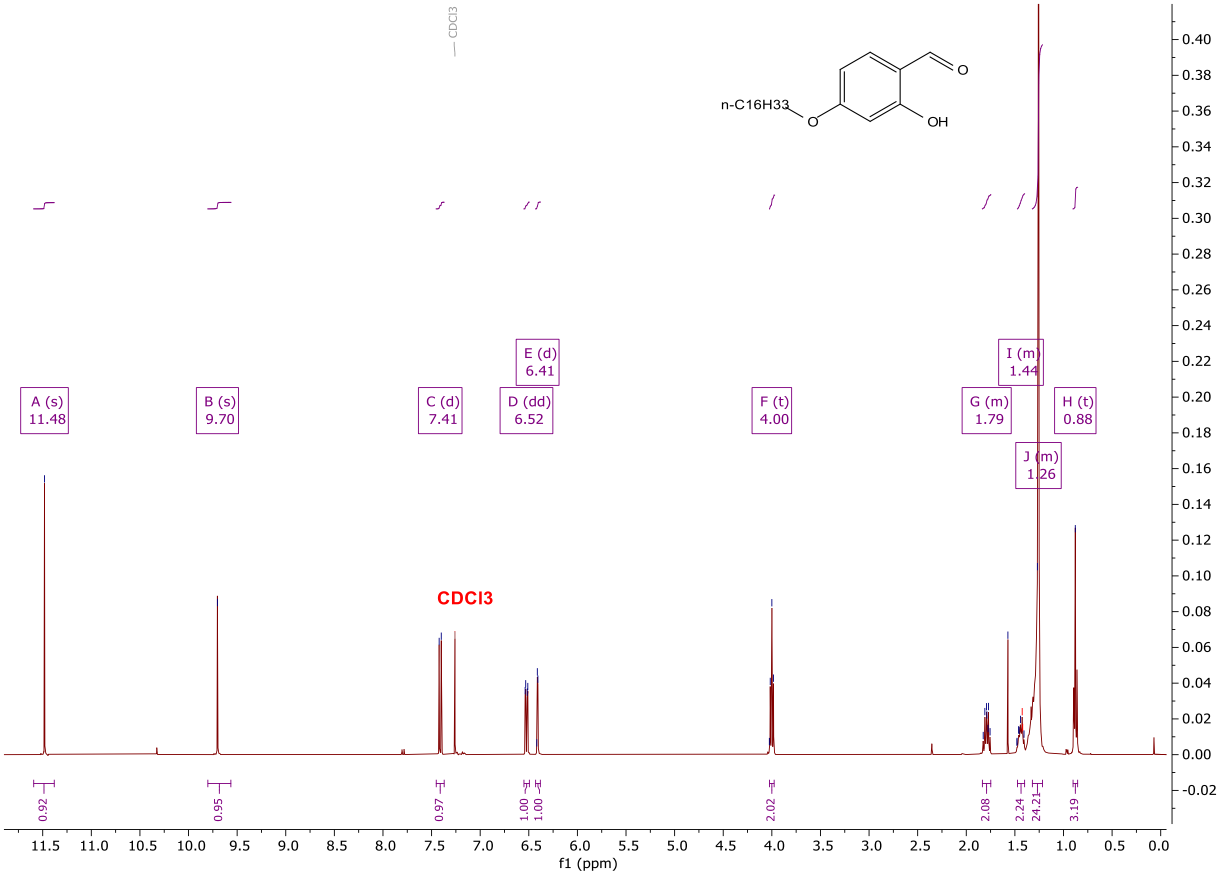

^{1}H NMR (400 MHz, $CDCl_3$) δ 11.48 (s, 1H), 9.70 (s, 1H), 7.41 (d, *J* = 8.7 Hz, 1H), 6.52 (dd, *J* = 8.7, 2.3 Hz, 1H), 6.41 (d, *J* = 2.3 Hz, 1H), 4.00 (t, *J* = 6.6 Hz, 2H), 1.83 – 1.75 (m, 2H), 1.47 – 1.40 (m, 2H), 1.32 – 1.22 (m, 24H), 0.88 (t, 3H).

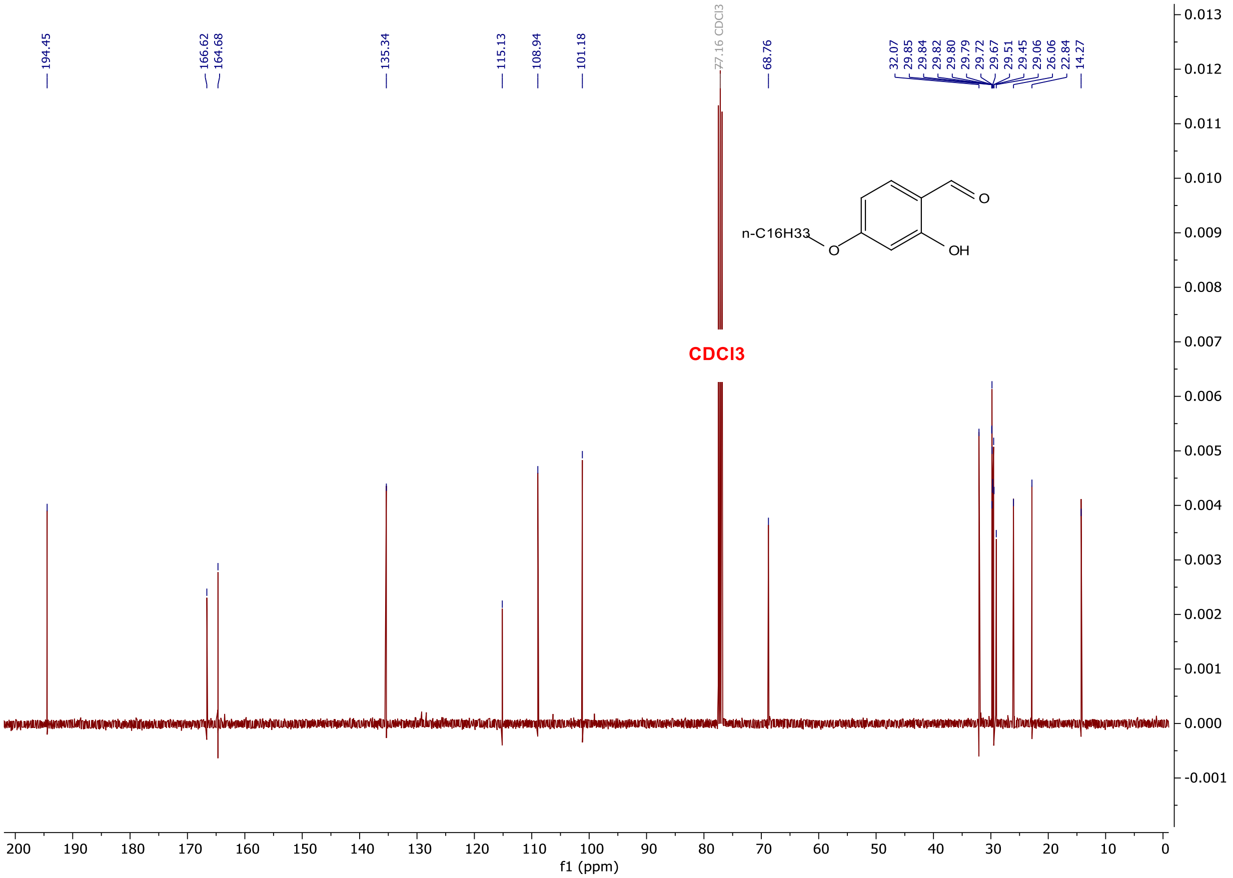

$^{13}C\{^1H\}$ NMR (101 MHz, $CDCl_3$) δ: 194.5, 166.6, 164.7, 135.3, 115.1, 108.9, 101.2, 68.8, 32.1, 29.9, 29.8, 29.8, 29.8, 29.8, 29.7, 29.7, 29.5, 29.5, 29.1, 26.1, 22.8, 14.3.*

*2.1.6 Synthesis of 5-(4-hexadecyloxy-2-hydroxybenzylidene)pyrimidine-2,4,6-trione (**3a**)*

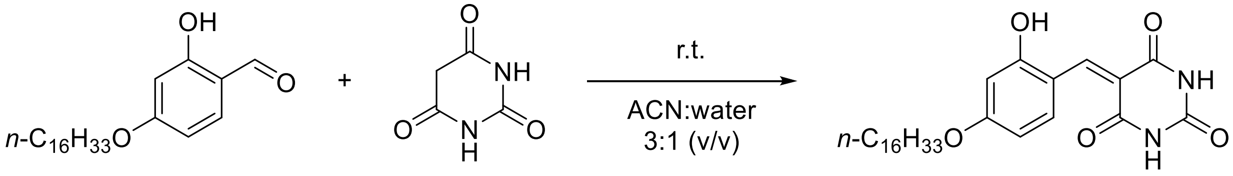

4-(Hexadecyloxy)-2-hydroxybenzaldehyde (0.16 g, 0.44 mmol) was dispersed in 15 mL of methanol and heated until all solid dissolved. While still hot, barbituric acid (0.056 g, 0.44 mmol) was added to the reaction mixture, which was stirred for 24 hours and cooled to r.t. Product **3a** was isolated by filtration as a deep-yellow solid. Yield = 97 % (0.20 g, 0.42 mmol).

* The signals which appear as identical chemical shift are resolved on the second decimal: 29.84, 29.82, 29.80, 29.79, 29.72, 29.67, 29.51, 29.45

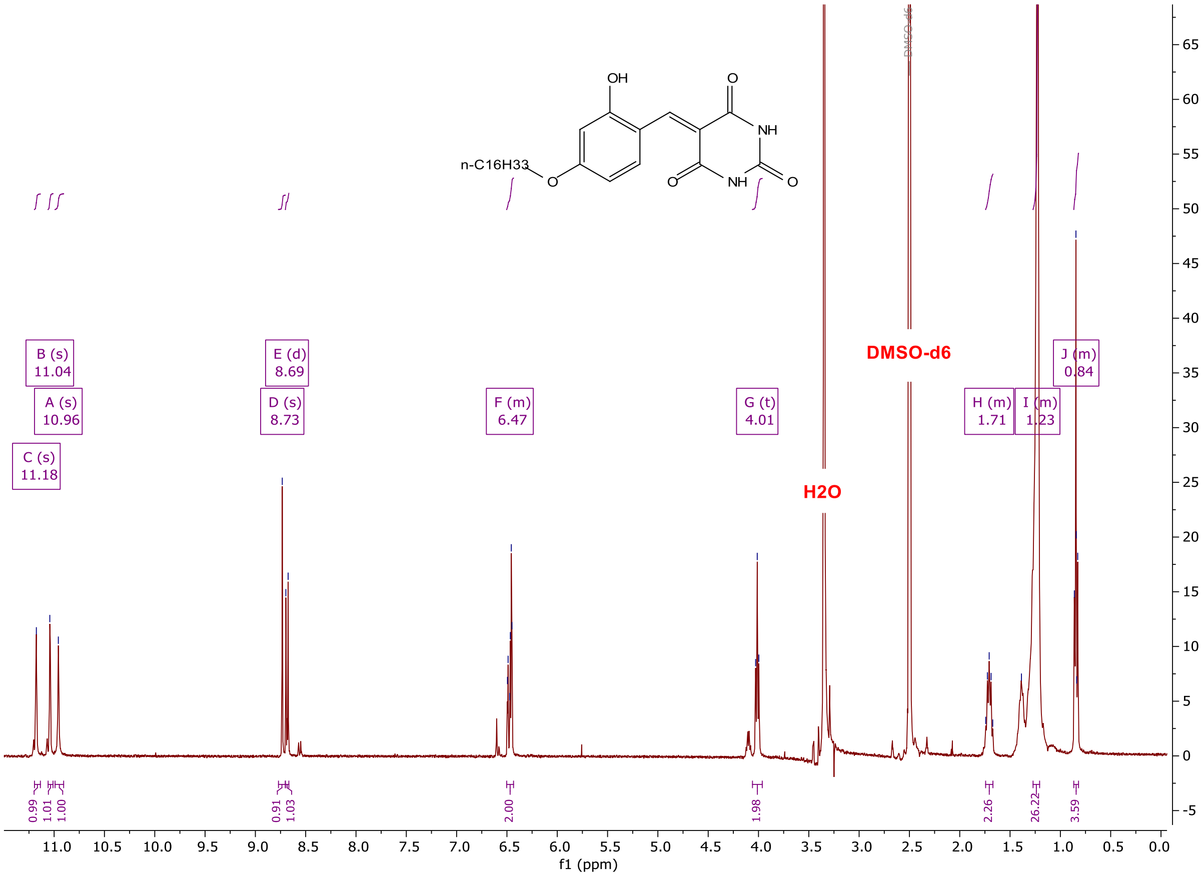

^{1}H NMR (400 MHz, DMSO-d_6) δ 11.18 (s, 1H), 11.04 (s, 1H), 10.96 (s, 1H), 8.73 (s, 1H), 8.69 (d, *J* = 9.0 Hz, 1H), 6.50 – 6.43 (m, 2H), 4.01 (t, *J* = 6.4 Hz, 2H), 1.74 – 1.67 (m, 2H), 1.27 – 1.21 (m, 26H), 0.87 – 0.82 (m, 4H).

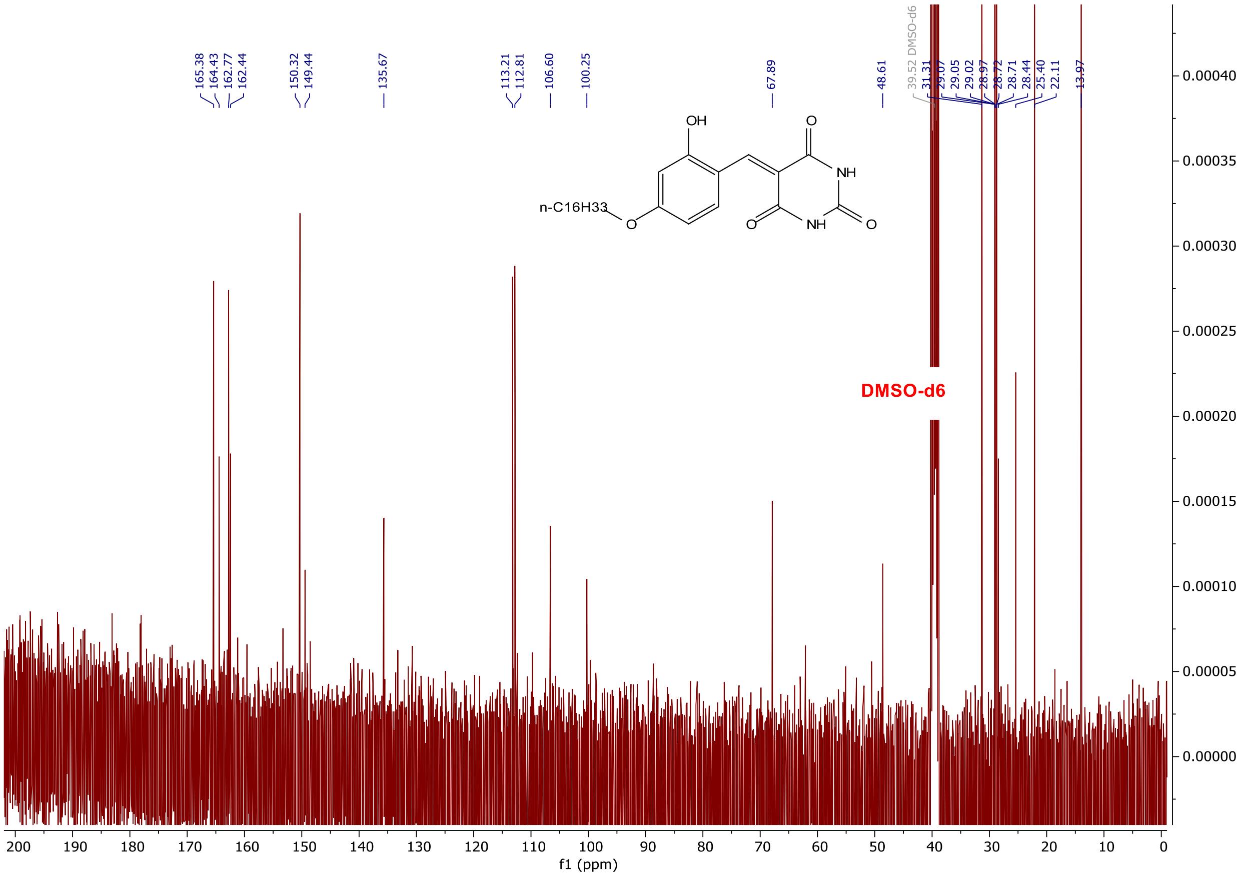

$^{13}C\{^{1}H\}$ NMR (101 MHz, DMSO-d_6) δ: 165.4, 164.4, 162.8, 162.4, 150.3 149.4, 135.7, 113.2, 112.8, 106.6, 100.3, 67.9, 48.6, 31.3, 29.1, 29.1, 29.0, 29.0, 28.7, 28.7, 28.4, 25.4, 22.1, 14.0.†

HRMS (ESI-) m/z $[M - H]^-$: calculated for $C_{27}H_{39}N_2O_5$ 471.2865, found 471.2869

*2.1.7 Synthesis of 8-hexadecyloxy-2H-chromeno[2,3-d]pyrimidine-2,4-dione (**3b**)*

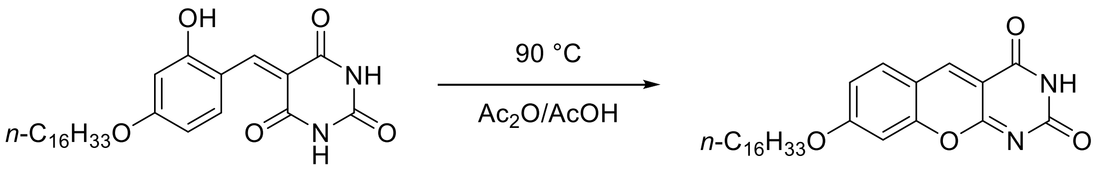

The cyclization of **3a** (0.20 g, 0.42 mmol) was carried out in 10 mL of a mixture of acetic acid and acetic anhydride (8:2 v/v) at 90 °C for 14 hours on a hot plate with heating mantle. After cooling to r.t., 20 mL of water was added, and the precipitate was collected by filtration, washed with water, and dried under vacuum. Product **3b** was obtained as a yellow powder (95 %, 0.18 g, 3.3 mmol).

† The signals which appear as identical chemical shift are resolved on the second decimal: 29.07, 29.05, 29.02, 28.97, 28.72, 28.71

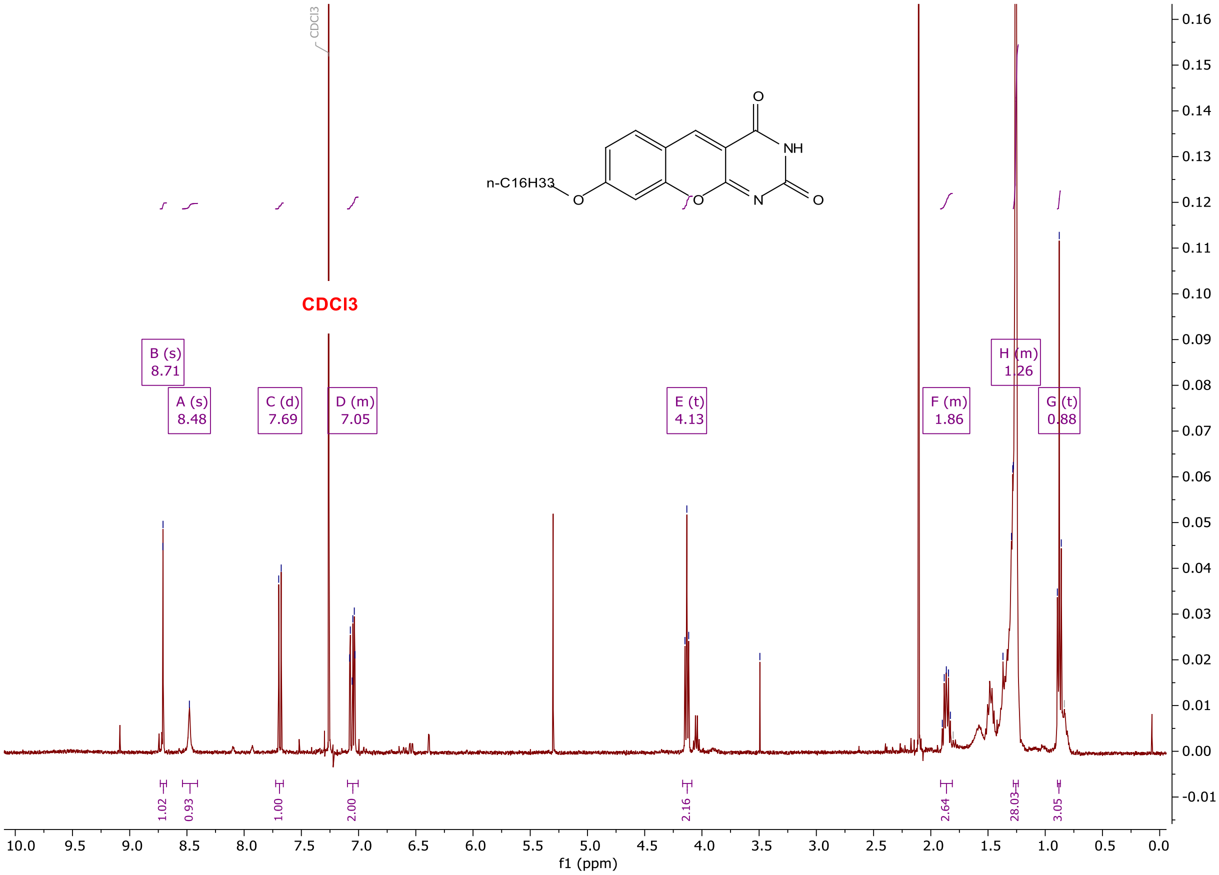

^{1}H NMR (400 MHz, C DCl_3) δ 8.71 (s, 1H), 8.48 (s, 1H), 7.69 (d, *J* = 8.7 Hz, 1H), 7.10 – 7.00 (m, 2H), 4.13 (t, *J* = 6.5 Hz, 2H), 1.91 – 1.81 (m, 3H), 1.28 – 1.24 (m, 28H), 0.88 (t, 3H).

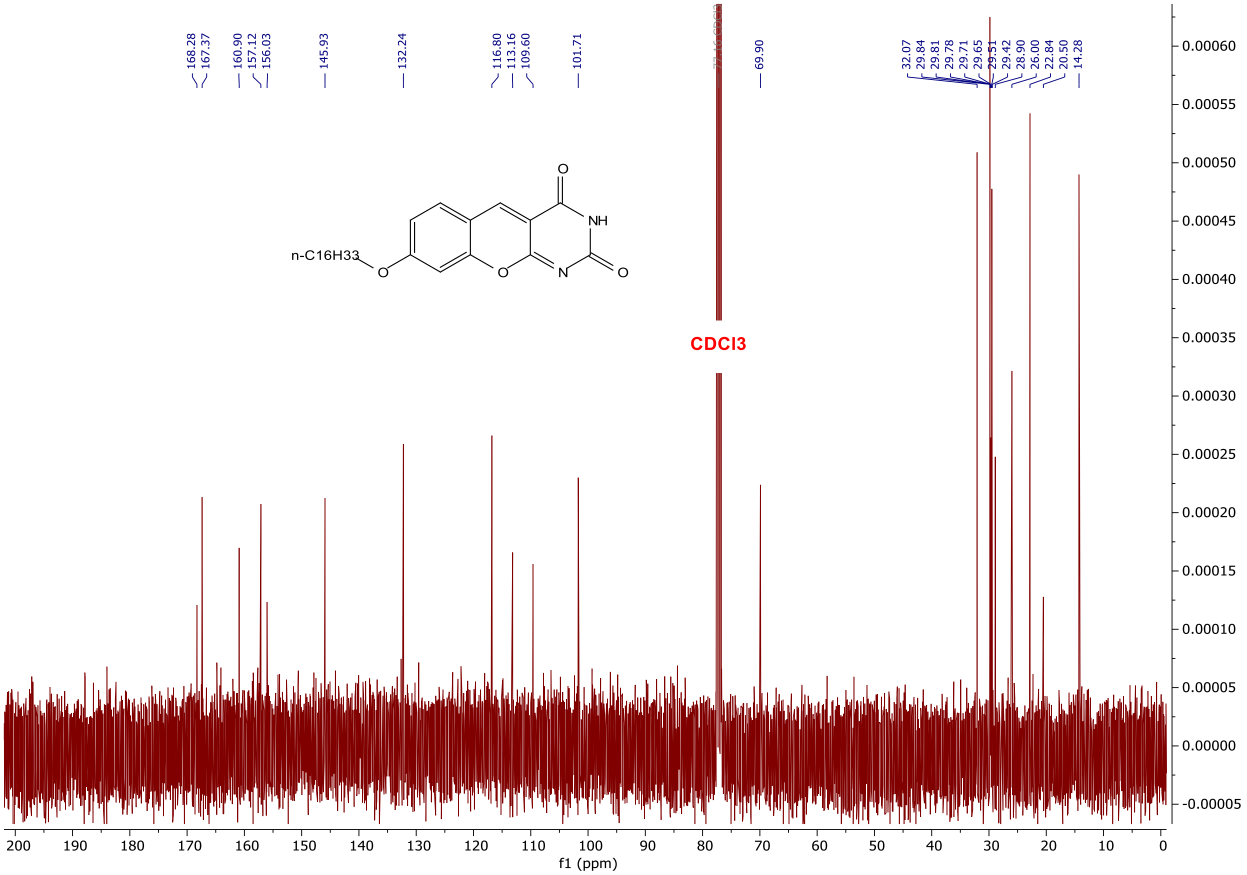

$^{13}C\{^{1}H\}$ NMR (101 MHz, $CDCl_3$) δ: 168.3, 167.4, 160.9, 157.1, 156.0, 145.9, 132.2, 116.8, 113.2, 109.6, 101.7, 69.9, 32.1, 29.8, 29.8, 29.8, 29.7, 29.7, 29.5, 29.4, 28.9, 26.0, 22.8, 20.5, 14.3.‡

HRMS (ESI-) m/z $[M - H]^-$: calculated for $C_{27}H_{37}N_2O_4$ 453.2759, found 453.2768

*2.1.8 Synthesis of 5-(2,4-dihydroxybenzylidene)pyrimidine-2,4,6-trione (**4a**)*

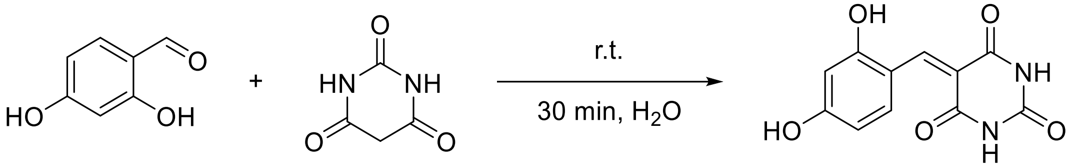

1.00 g of barbituric acid was dissolved in 20 mL of water. Then, 1.08 g (7.8 mmol) of 2,4-dihydroxybenzaldehyde was added. The reaction mixture quickly turned to yellow suspension. After stirring for 30 minutes at r.t., the suspension was filtered and the precipitate was washed by water and ethanol. Drying under high vacuum afforded **4a** in 90 % yield (1.74 g).

‡ The signals which appear as identical chemical shift are resolved on the second decimal: 29.84, 29.81, 29.78, 29.71, 29.65

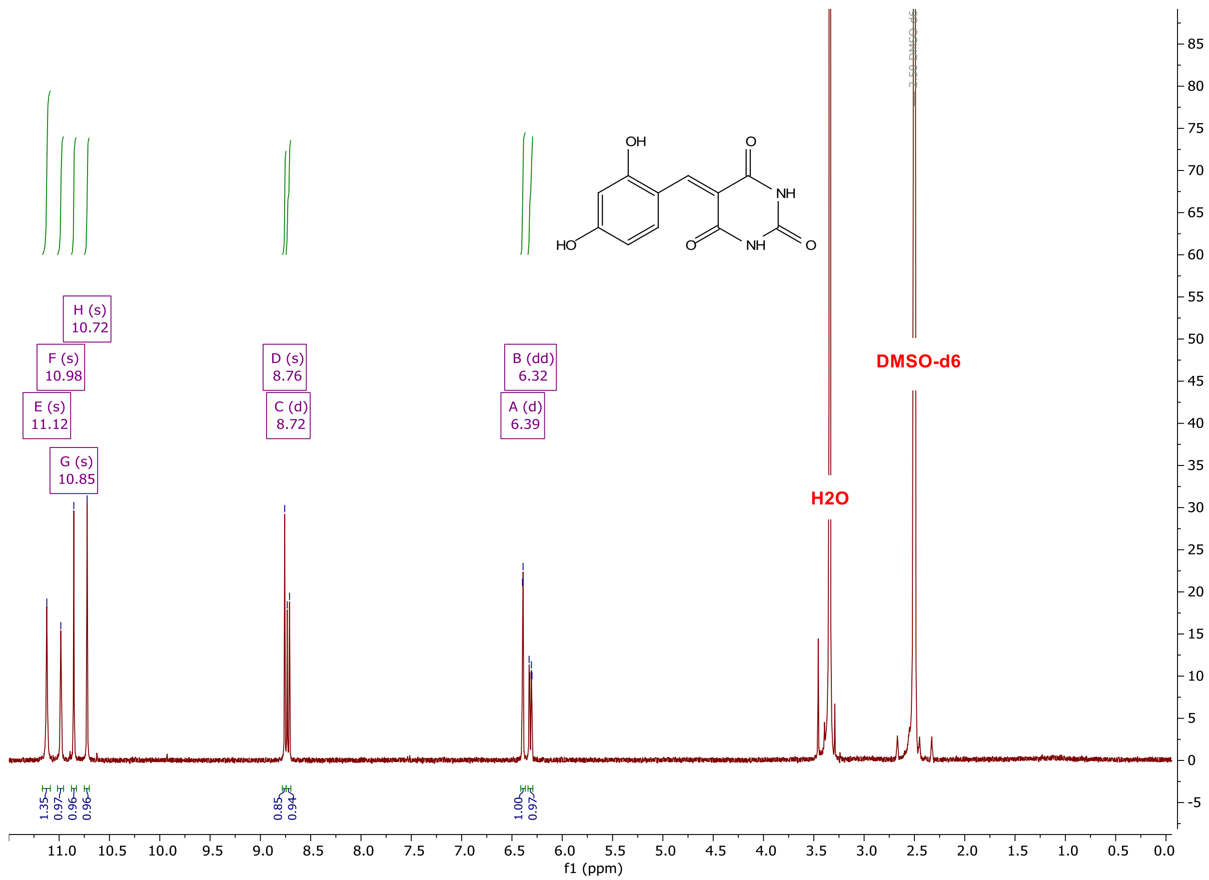

^{1}H NMR (400 MHz, DMSO-d_6) δ: 11.12 (s, 1H), 10.98 (s, 1H), 10.85 (s, 1 H), 10.72 (s, 1H), 8.76 (s, 1H), 8.72 (d, J = 9.1 Hz, 1H), 6.39 (d, J = 2.4 Hz, 1H), 6.32 (dd, J = 9.1, 2.4 Hz, 1H).

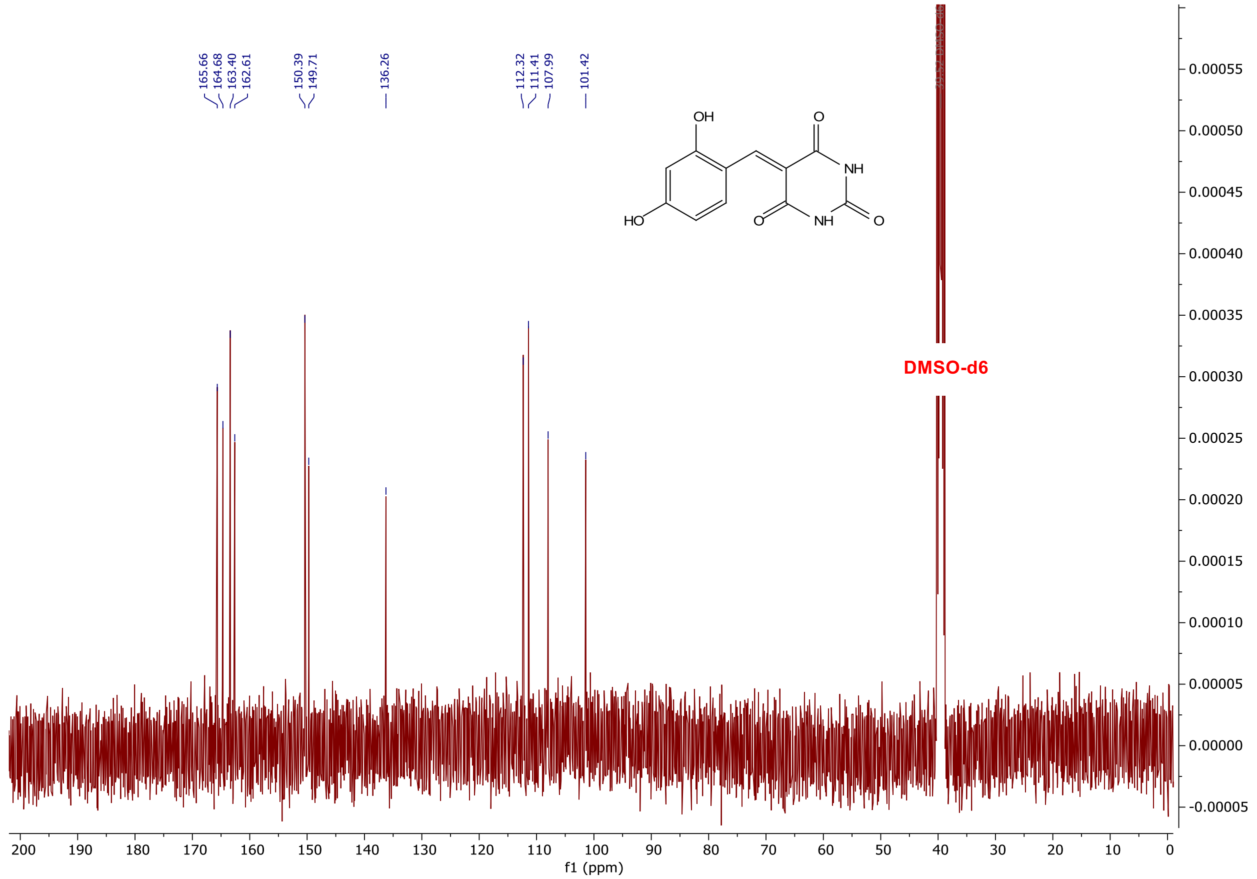

$^{13}C\{^1H\}$ NMR (101 MHz, DMSO-d_6) δ: 165.7, 164.7, 163.4, 162.6, 150.4, 149.7, 136.3, 112.3, 111.4, 108.0, 101.4.

HRMS (ESI-) m/z [M - H]$^-$: calculated for $C_{11}H_7N_2O_5$ 247.0360, found 247.0361

2.1.9 *Synthesis of 8-hydroxy-2H-chromeno[2,3-d]pyrimidine-2,4-dione (**4b**)*

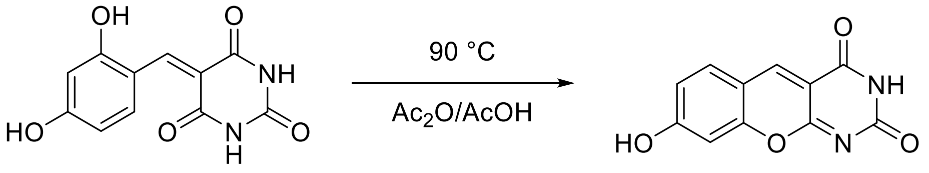

1.74 g of 4a was suspended in a mixture of acetic acid and acetic anhydride (8:2 v/v) and heated at 90 °C under reflux condenser for 90 minutes on a hot plate with heating mantle. After the suspension cooled down to r.t., it was filtered and the precipitate was washed by ethyl acetate. After drying under high vacuum, product **4b** was isolated in 84 % yield (1.51 g) as a yellow powder.

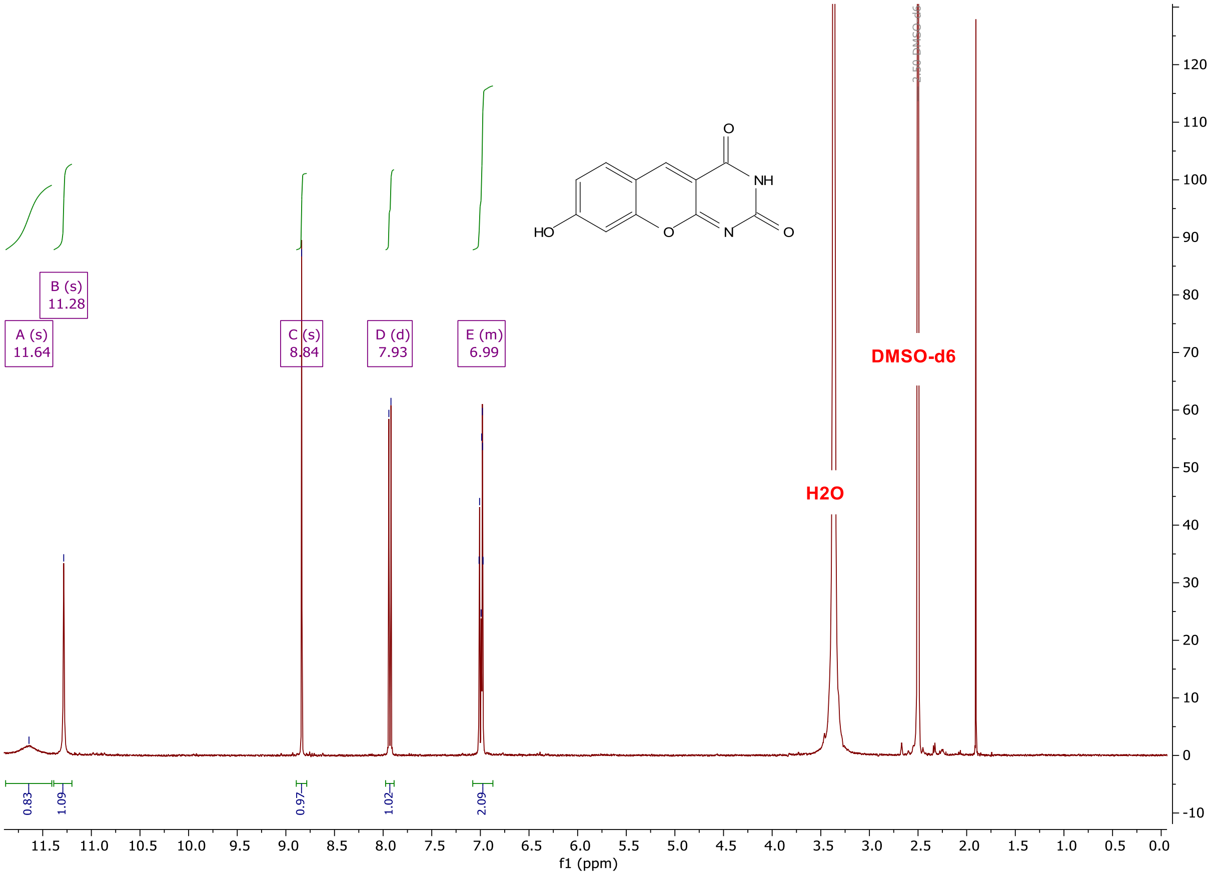

^{1}H NMR (400 MHz, DMSO-d_6) δ: 11.64 (br s, 1H), 11.28 (s, 1H), 8.84 (s, 1H), 7.93 (d, *J* = 8.6 Hz, 1H), 7.04 – 6.95 (m, 2H).

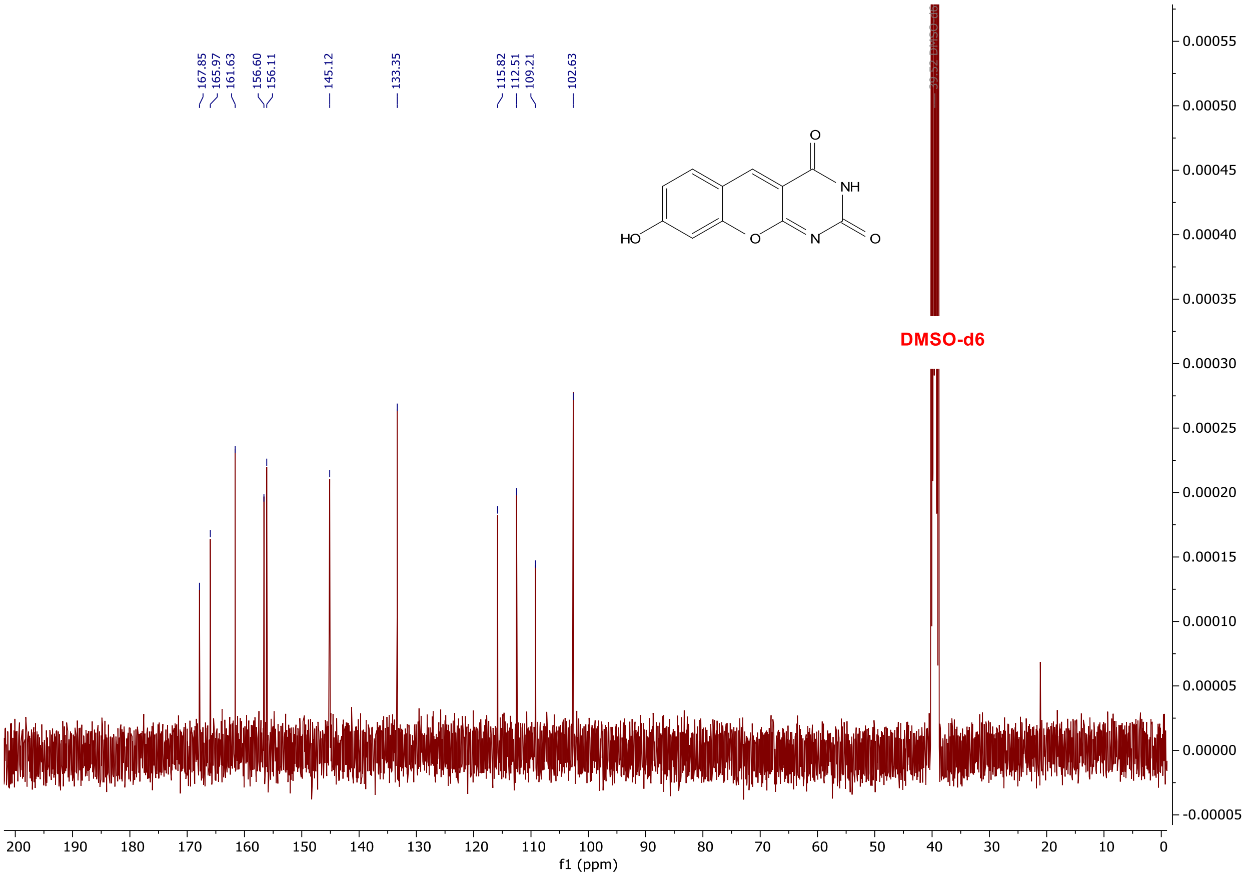

$^{13}C\{^1H\}$ NMR (101 MHz, DMSO-d_6) δ: 167.9, 166.0, 161.6, 156.6, 156.1, 145.1, 133.4, 115.8, 112.5, 109.2, 102.6.

HRMS (ESI-) m/z $[M - H]^-$: calculated for $C_{11}H_5N_2O_4$ 229.0255, found 229.0253

*2.1.10 Synthesis of 5-(2-hydroxy-3-methoxybenzylidene)pyrimidine-2,4,6-trione (**5a**)*

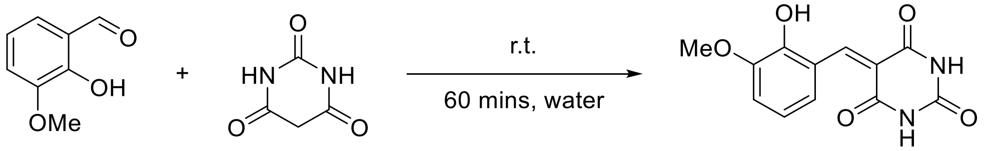

2-Hydroxy-3-methoxybenzaldehyde (0.50 g, 3.28 mmol) and barbituric acid (0.42 g, 2.38 mmol) were mixed in 10 mL of water and stirred for 60 minutes at r.t., during which the reaction mixture turned into yellow suspension. It was then filtered and the solid was washed with water and ethanol. After drying under high vacuum, it was found that the crude product (0.70 g) contained unreacted starting aldehyde (*ca* 10 % mol). This impurity was impossible to remove by excessive washing. The compounds were also found inseparable by column chromatography and were thus subjected to the following step as a mixture of species. NMR signals reported below are assigned based on known shifts of starting compounds and final product **5b**.

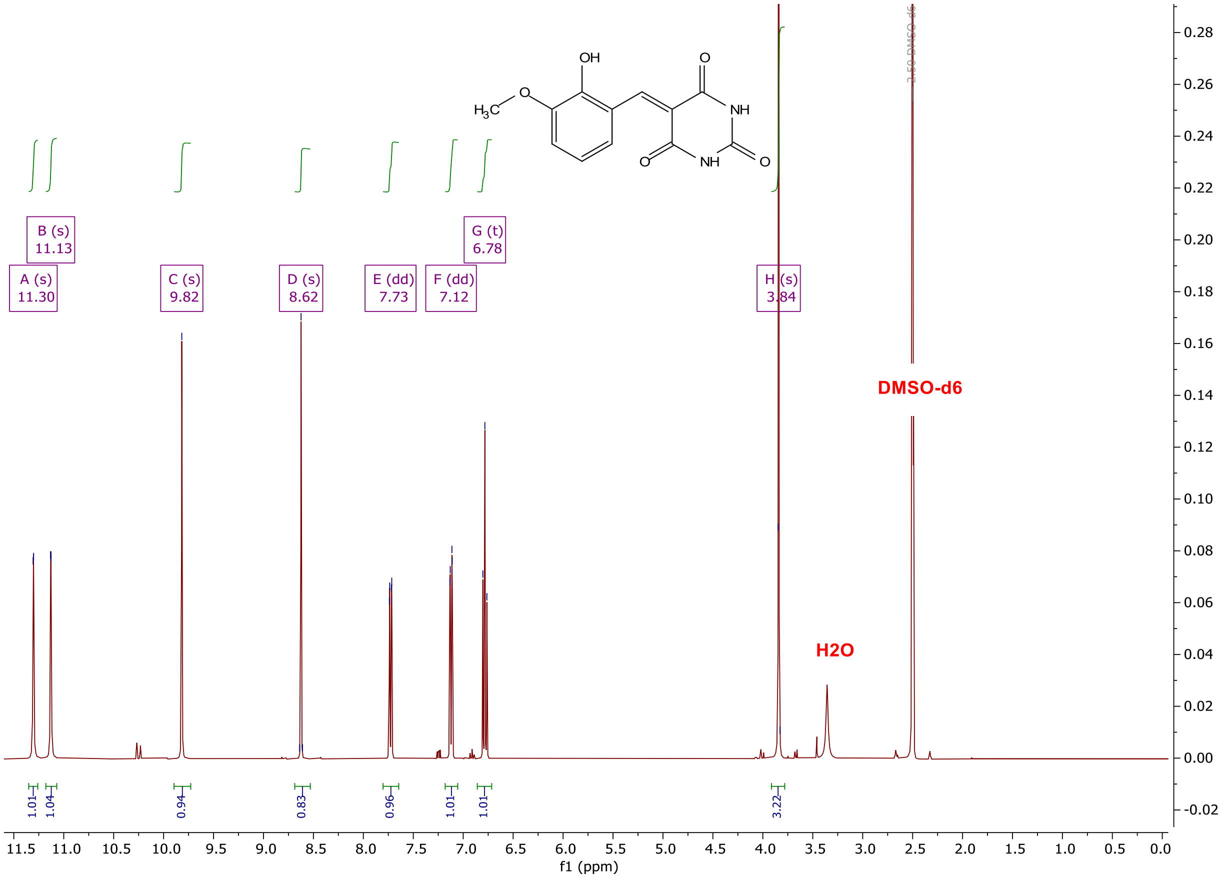

^{1}H NMR (400 MHz, DMSO-d_6) δ: 11.30 (s, 1H), 11.13 (s, 1H), 9.82 (s, 1H), 8.62 (s, 1H), 7.73 (dd, *J* = 8.2, 1.4 Hz, 1H), 7.12 (dd, *J* = 8.1, 1.4 Hz, 1H), 6.78 (t, *J* = 8.0 Hz, 1H), 3.84 (s, 3H).

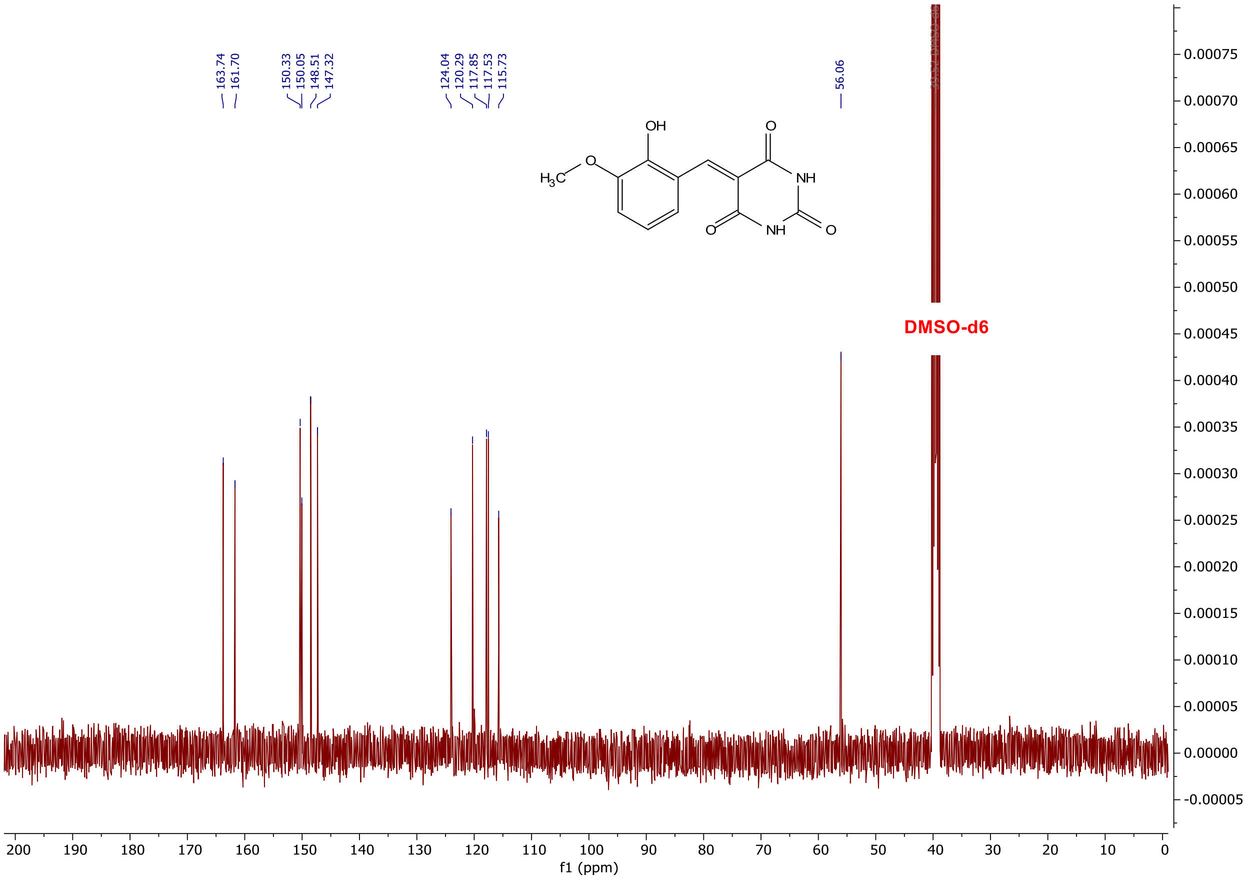

$^{13}C\{^{1}H\}$ NMR (101 MHz, DMSO-d_6) δ: 163.7, 161.7, 150.3, 150.1, 148.5, 147.3, 124.0, 120.3, 117.9, 117.5, 115.7, 56.1.

HRMS (ESI-) m/z $[M - H]^-$: calculated for $C_{12}H_7N_2O_4$ 261.0517, found 261.0512

*2.1.11 Synthesis of 9-methoxy-2H-chromeno[2,3-d]pyrimidine-2,4-dione (**5b**)*

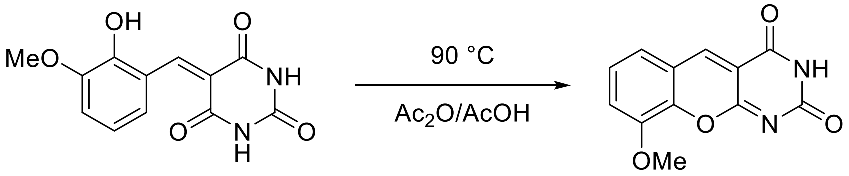

The crude product (0.70 g) **5a** containing 10 mol % of starting aldehyde as inseparable impurity was heated at 90 °C in a mixture of AcOH/Ac_2O (8:2 v/v) for 90 minutes. After cooling down, the product precipitate as a yellow solid, which was collected by filtration and washed by ethyl acetate. Drying in high vacuum afforded 0.46 g of **5b** (75 % yield).

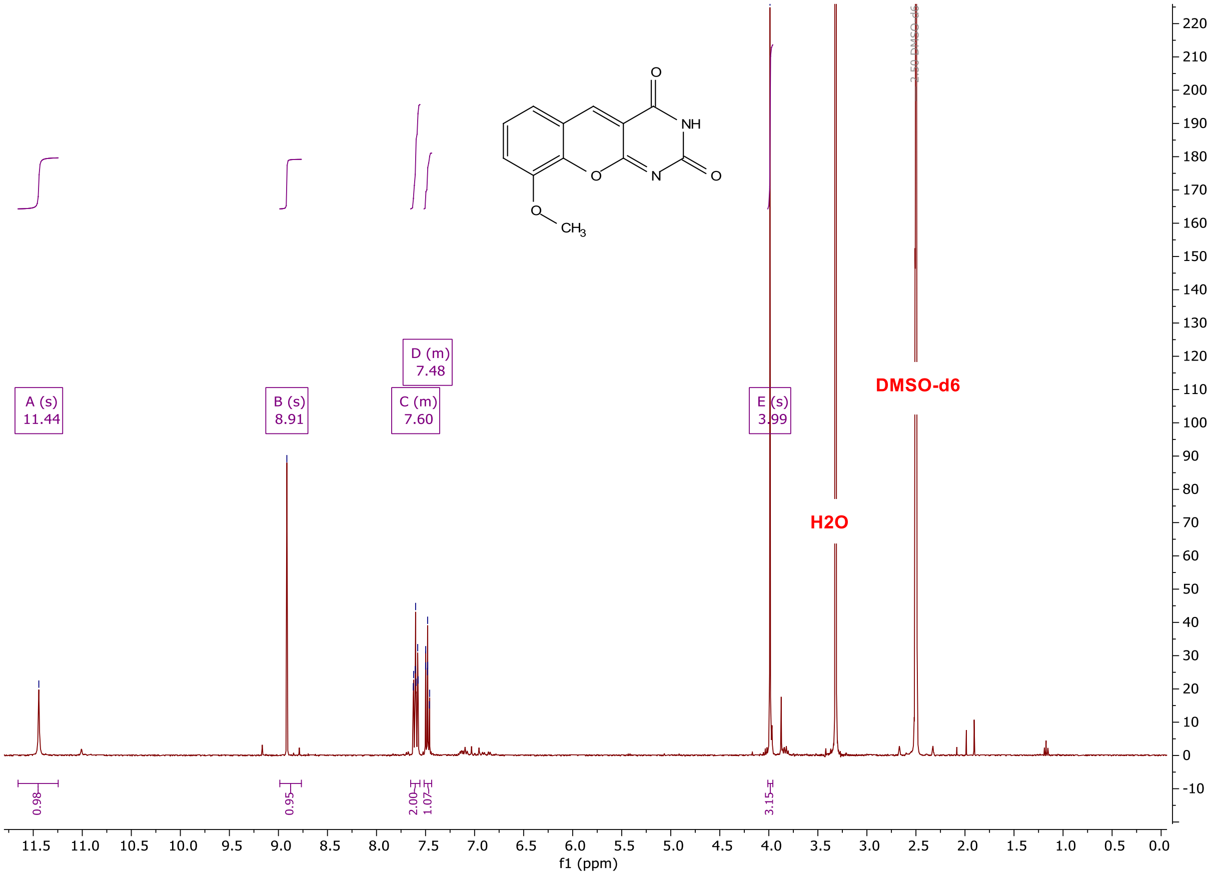

^{1}H NMR (400 MHz, DMSO-*d*$_6$) δ 11.44 (s, 1H), 8.91 (s, 1H), 7.65 – 7.56 (m, 2H), 7.51 – 7.44 (m, 1H), 3.99 (s, 3H).

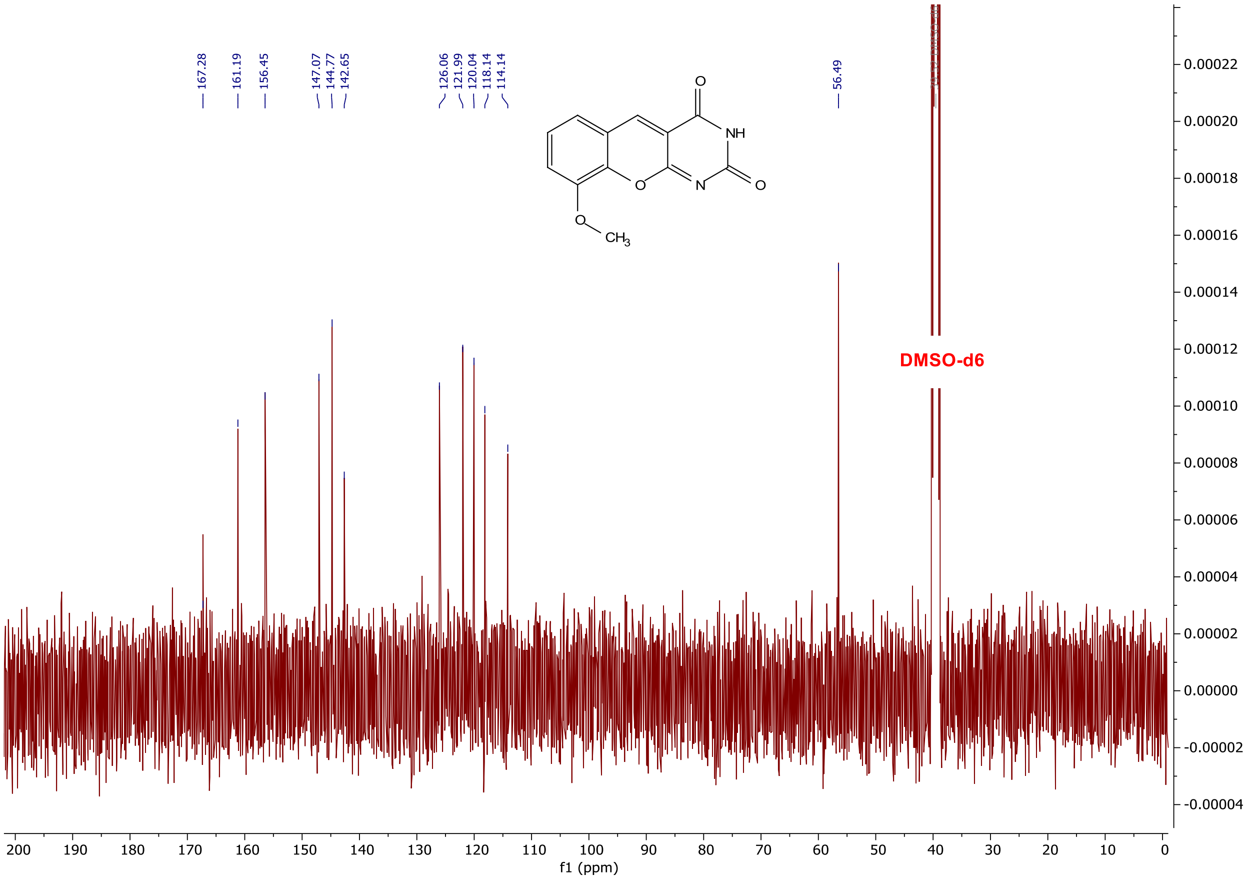

$^{13}C\{^{1}H\}$ NMR (101 MHz, DMSO-d_6) δ: 167.3, 161.2, 156.5, 147.1, 144.8, 142.7, 126.1, 122.0, 120.0, 118.1, 114.1, 56.5.

HRMS (ESI-) m/z $[M - H]^-$: calculated for $C_{12}H_7N_2O_4$ 243.0411, found 243.0410

*2.1.12 Synthesis of 5-(2-hydroxy-5-methoxybenzylidene)pyrimidine-2,4,6-trione (**6a**)*

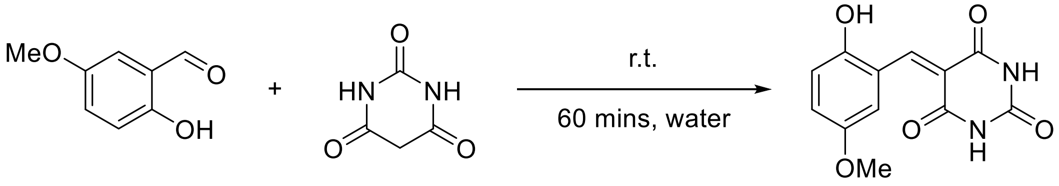

In 10 mL of water, 0.50 mL (4.0 mmol) of 2-hydroxy-5-methoxybenzaldehyde and 0.51 g (4.0 mmol) of barbituric acid were mixed and stirred at r.t. for 60 minutes. The reaction mixture turned into a deep red suspension. Then, it was filtered, the solid was washed by water and ethanol and dried under high vacuum. **6a** was isolated as a red powder in 86 % yield (0.90 g).

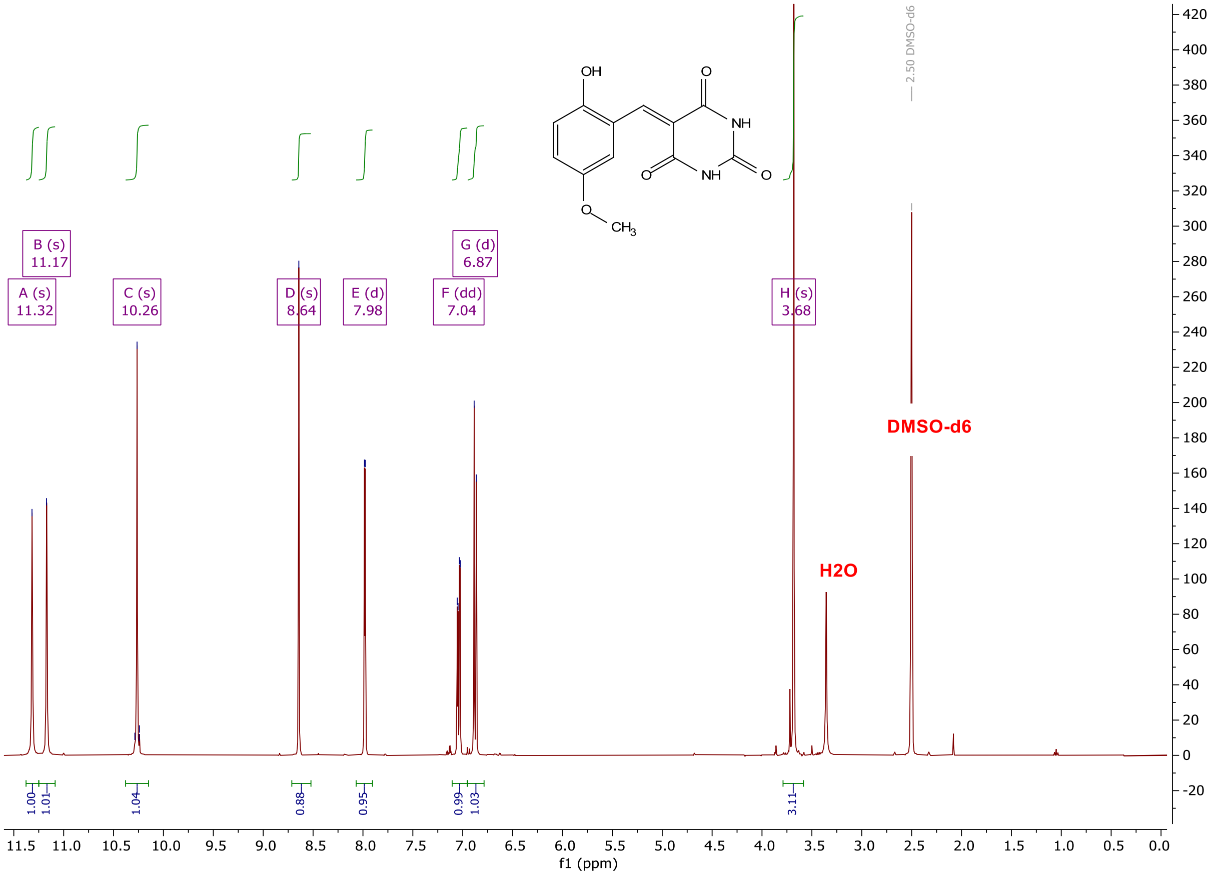

^{1}H NMR (400 MHz, DMSO-d_6) δ: 11.32 (s, 1H), 11.17 (s, 1H), 10.26 (s, 1H), 8.64 (s, 1H), 7.98 (d, J = 3.2 Hz, 1H), 7.04 (dd, J = 8.9, 3.2 Hz, 1H), 6.87 (d, J = 9.0 Hz, 1H), 3.68 (s, 3H).

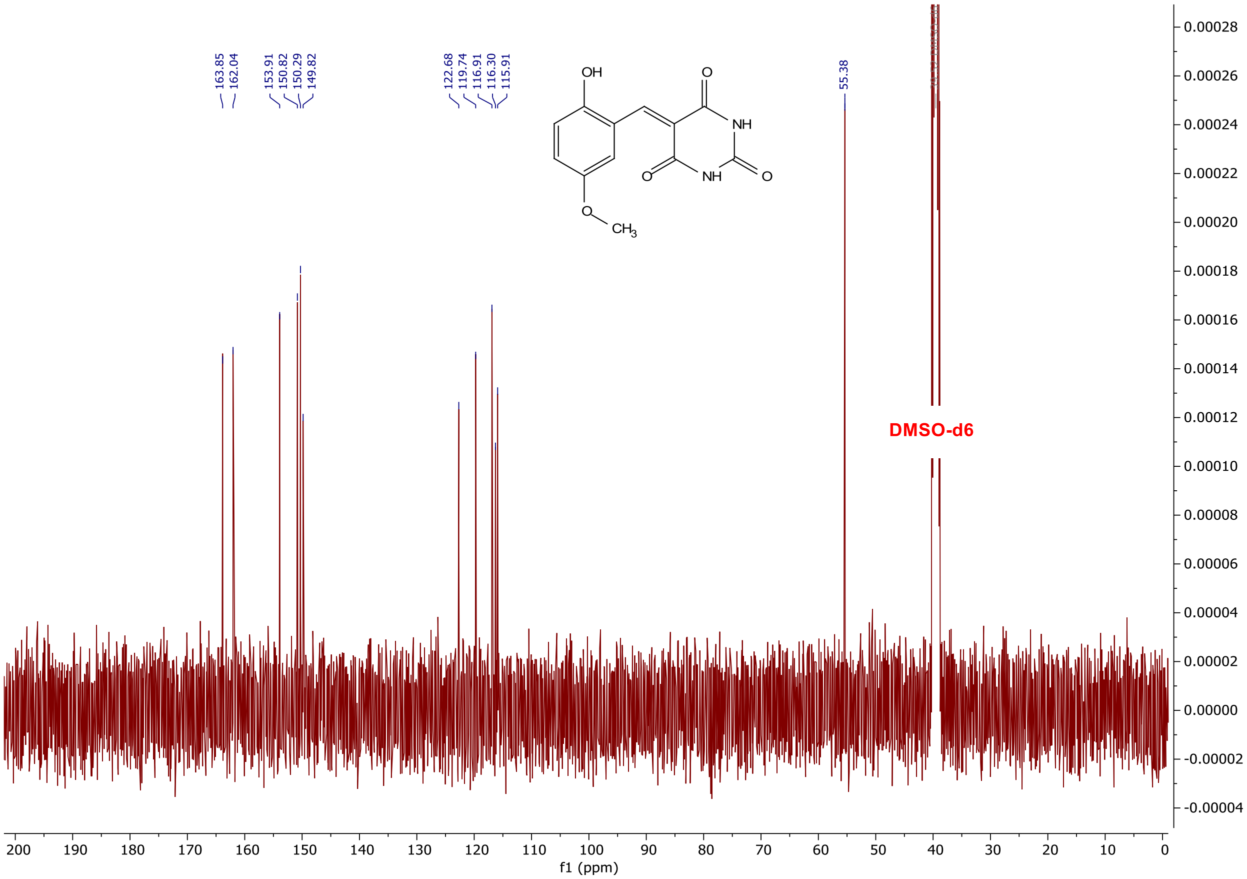

$^{13}C\{^{1}H\}$ NMR (101 MHz, DMSO-d_6) δ: 163.9, 162.0, 153.9, 150.8, 150.3, 149.8, 122.7, 119.7, 116.9, 116.3, 115.9, 55.4.

HRMS (ESI-) m/z $[M - H]^-$: calculated for $C_{12}H_9N_2O_5$ 261.0517, found 261.0513

2.1.13 *Synthesis of 7-methoxy-2H-chromeno[2,3-d]pyrimidine-2,4-dione (**6b**)*

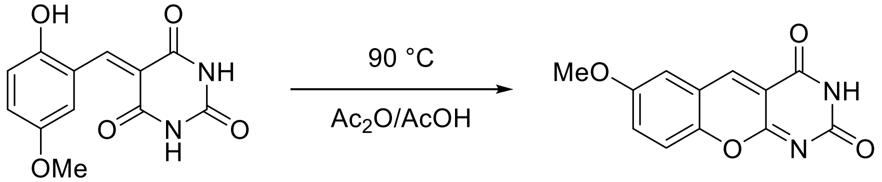

Intermediate **6a** (0.90 g, 3.38 mmol) was stirred in 20 mL of a mixture of acetic acid and acetic anhydride (8:2 v/v) at 90 °C on a hot plate with heating mantle. for 90 minutes under reflux condenser, gradually decolorizing to yellow. The reaction mixture was then allowed to cool down to r.t. Filtration, washing by ethyl acetate, and drying under high vacuum afforded 0.72 g of the product **6b** (86 %) as a yellow powder.

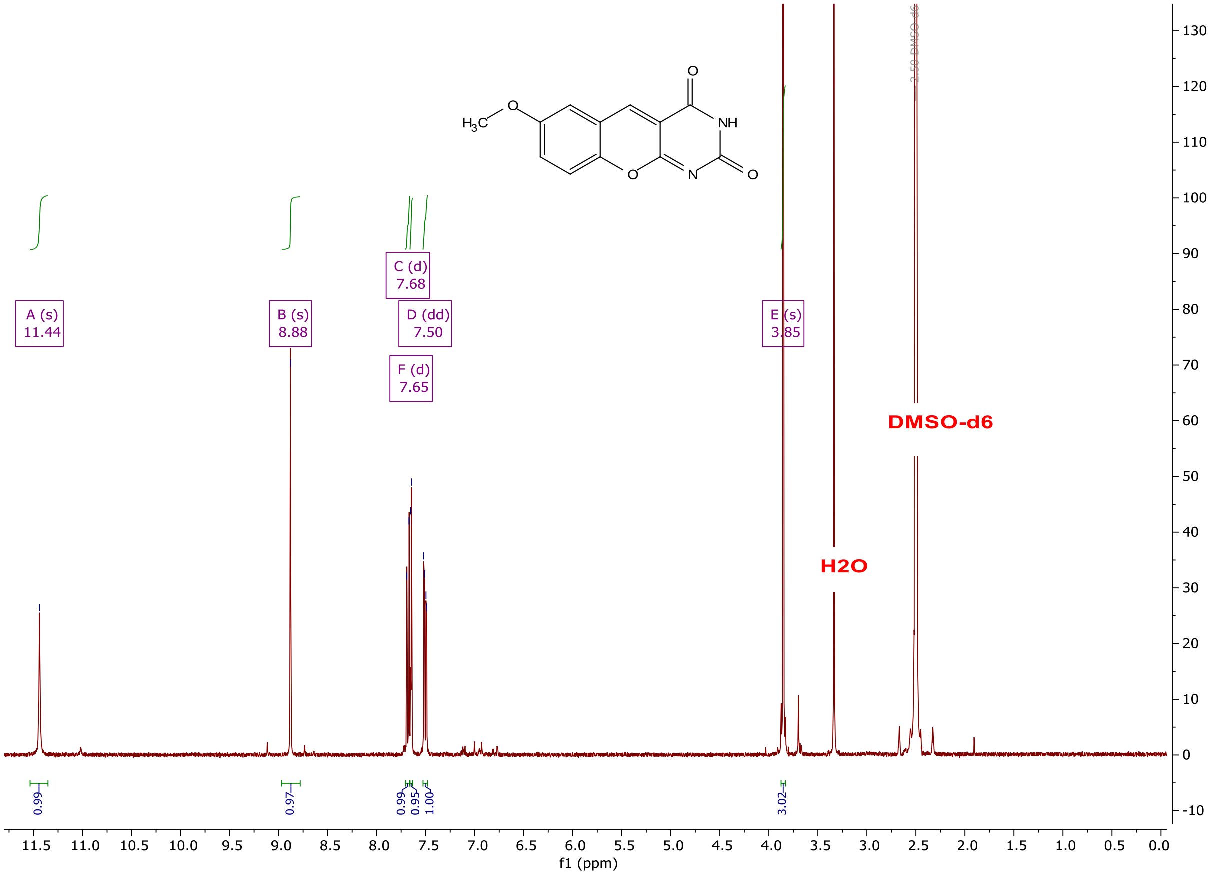

^{1}H NMR (400 MHz, DMSO-d_6) δ 11.44 (s, 1H), 8.88 (s, 1H), 7.68 (d, *J* = 9.2 Hz, 1H), 7.65 (d, *J* = 3.0 Hz, 1H), 7.50 (dd, *J* = 9.1, 3.1 Hz, 1H), 3.85 (s, 3H).

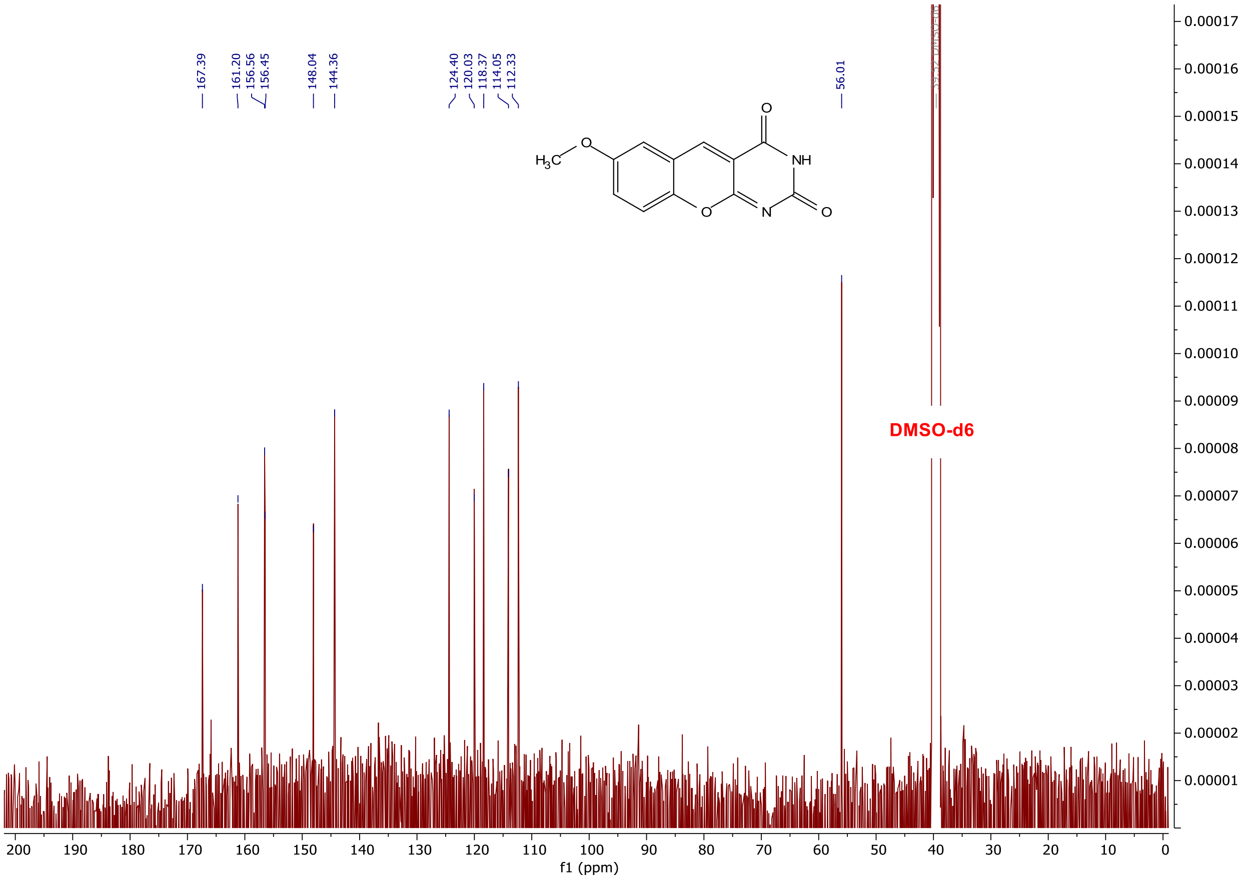

$^{13}C\{^1H\}$ NMR (101 MHz, DMSO-d_6) δ: 167.4, 161.2, 156.6, 156.5, 148.0, 144.4, 124.4, 120.0, 118.4, 114.1, 112.3, 56.0.

HRMS (ESI-) m/z $[M - H]^-$: calculated for $C_{12}H_7N_2O_4$ 243.0411, found 243.0411

3 Determination of HOMO and LUMO energies

Cyclic voltammetry (CV) and UV-vis spectroscopy were performed to estimate the HOMO and LUMO energy levels. The CV measurements were performed as follows: 2 × 10^{-3} M solutions of analytes **b** and TARF were prepared in dry *N,N*-dimethylformamide (DMF, acetonitrile could not have been used due to insufficient solubility). Tetrabutylammonium hexafluorophosphate (Bu_4NPF_6) was added as a supporting electrolyte to reach 0.05 M concentration in the solution. The measurements were performed using Ag/AgCl as the pseudoreference electrode, Pt wire as the counter electrode and Pt mesh as the working electrode. All measurements were carried out at room temperature in the potential range between -2 and 1.5 V with a scan rate of 50 mV/s. In this work, the ferrocenium/ferrocene (Fc^+/Fc) redox couple was used as an internal reference to calibrate our pseudoreference electrode (0.4 V *vs* Ag/AgCl).[31,32]

The LUMO energy was calculated from CV based on the difference from the ferrocene/ferrocenium redox couple with the value Fc_{HOMO} = -4.8 eV. This value is debated in the field.[33] To be consistent with related literature,[34] we adopt it here, as we only discuss the relative orbital energy differences, which are not influenced by the exact positioning of the electrochemical vs. the Fermi scales. Hence, with internal referencing of our pseudoreference electrode to Fc/Fc^+, we calculate $E_{LUMO} = -\left[4.8 - \left(E^{1/2}_{Fc/Fc^+} - E^{1/2}_{red}\right)\right](eV)$, where $E^{1/2}_{red}$ is the reduction potential of the molecule. It is worth mentioning that the prepared materials show stable behavior during repeated cycling (Figure S 1). The HUMO energies can be estimated from $E_{LUMO} - E_{HOMO} = E_{gap}(optical)$. $Egap\ (optical)$ is the absorbance onset determined from UV-vis spectra as an intercept of the tangent of the most bathochromically shifted absorbance feature with the λ-axis (Figure S 2). The relevant values of the energies (HOMO, LUMO, Energy gap) are given Table S 1.

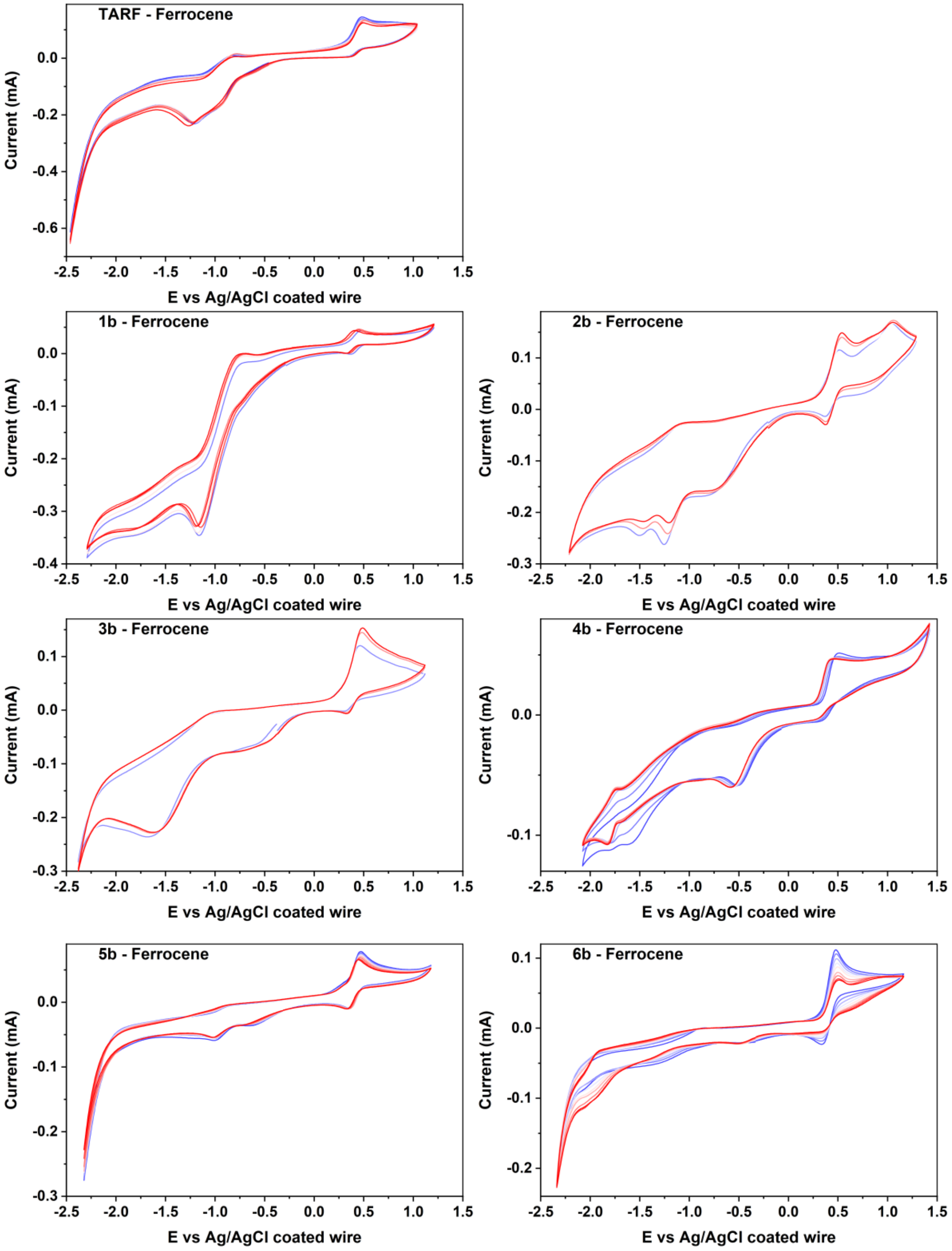

*Figure S 1 All the graphs follow the IUPAC convention. Cyclic voltammograms of the prepared molecules with Fc/Fc$^+$ internal standard: **TARF** and **1-6b** (analytes at 0.002 M conc.). CVs were recorded at a 50 mV/s sweep rate for 10 cycles in dry DMF containing 0.05 M electrolyte (Bu_4NPF_6) at room temperature. The measurements were performed using Ag/AgCl as the reference electrode, Pt wire as the counter electrode and Pt mesh as the working electrode. The voltammograms were collected, before correction, starting from 0 V and scanning towards negative potential first, then the data were corrected by calibrating respect to Fc/Fc$^+$ at 0.4 V.*

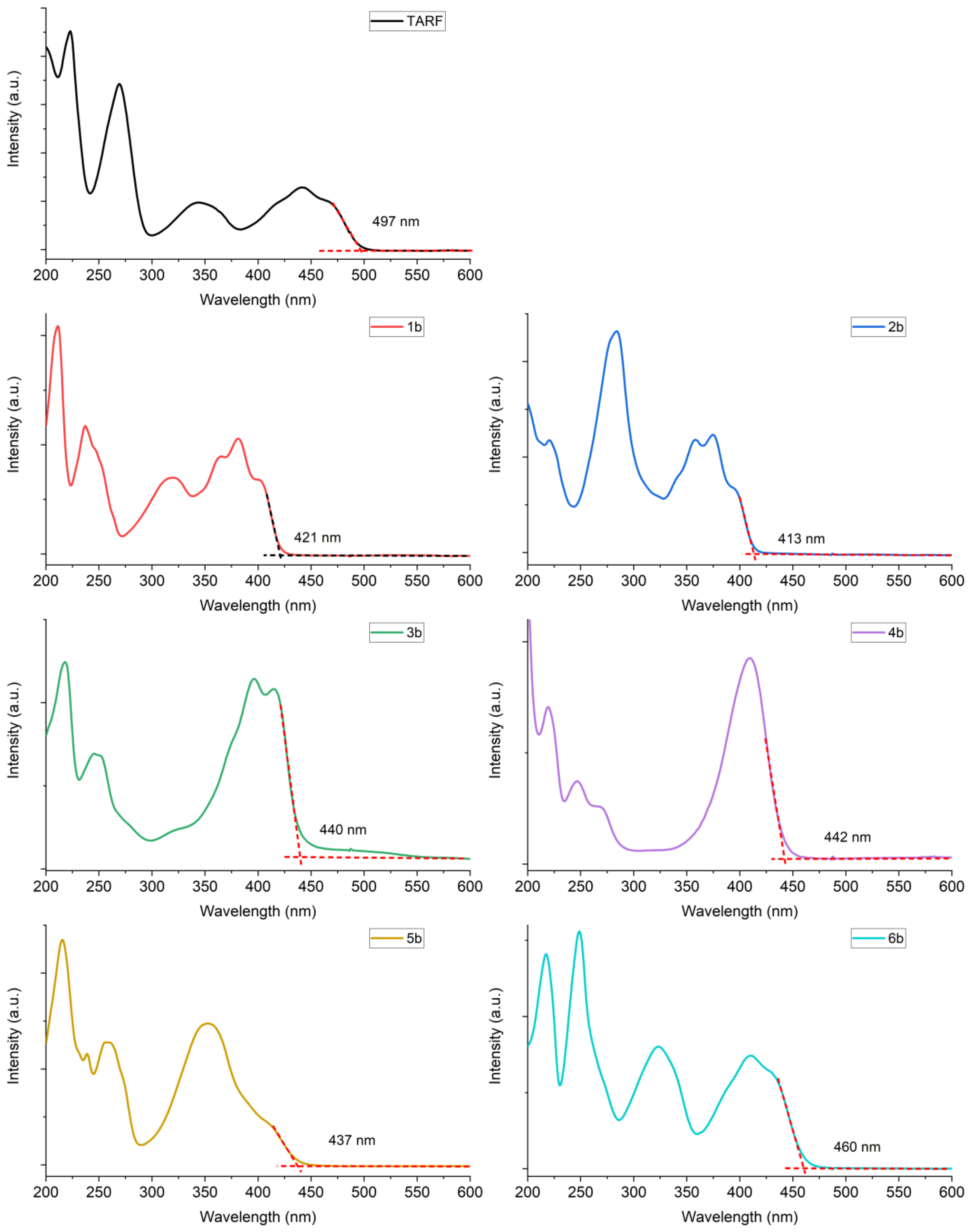

*Figure S 2 Absorption spectra of **TARF** and **1-6b** with the indicated determination of absorbance onset.*

*Table S 1 Summary of the calculated HOMO, LUMO and gap energies (Eg) of the molecules (TARF and **1-6b**) correlated with the photosulfoxidation quantum yield (ϕ_{ox}). The values for TARF are in good agreement with the literature.*[4,35–37]

Molecules	***E HOMO (eV)***	***E LUMO (eV)***	***Eg (eV)***	$\boldsymbol{\Phi_{ox}}$
TARF	-5.87	-3.38	2.49	0.18(ref 4)
1b	-6.40	-3.45	2.95	0.22
2b	-6.18	-3.18	3.00	0.03
3b	-5.91	-3.09	2.82	0.05
4b	-6.77	-3.96	2.81	0.65
5b	-6.30	-3.46	2.84	0.10
6b	-6.36	-3.66	2.70	0.18

The photooxidation quantum yields were calculated using the following equation:

$$\Phi_{ox} = \frac{molecules\ converted}{photons\ absorbed} = \frac{(c_0 - c_t)}{q_p \cdot t \cdot (1 - 10^{-A_{400nm}})}$$

where q_p is the photon flux in E s^{-1} cm^{-3}, c is concentration in mol cm^{-3}, and A_{400nm} is the absorbance of the reaction mixture in the vessel at 400 nm. The change in concentration during the first 300 seconds was used for all derivatives except 4b, which exhibited the fastest kinetics and the value in t=120 seconds was used instead.

4 Spectroscopy

To measure the absorbance and photoluminescence spectra of final derivatives **b** and TARF, solutions were prepared in UV grade acetonitrile each with a known concentration (all comprised between 2.4 and 3.5 × 10^{-5} M). The Beer–Lambert law $\varepsilon = \frac{A}{cd}$

was used to determine the extinction coefficient, where d = 1.00 cm in our system.

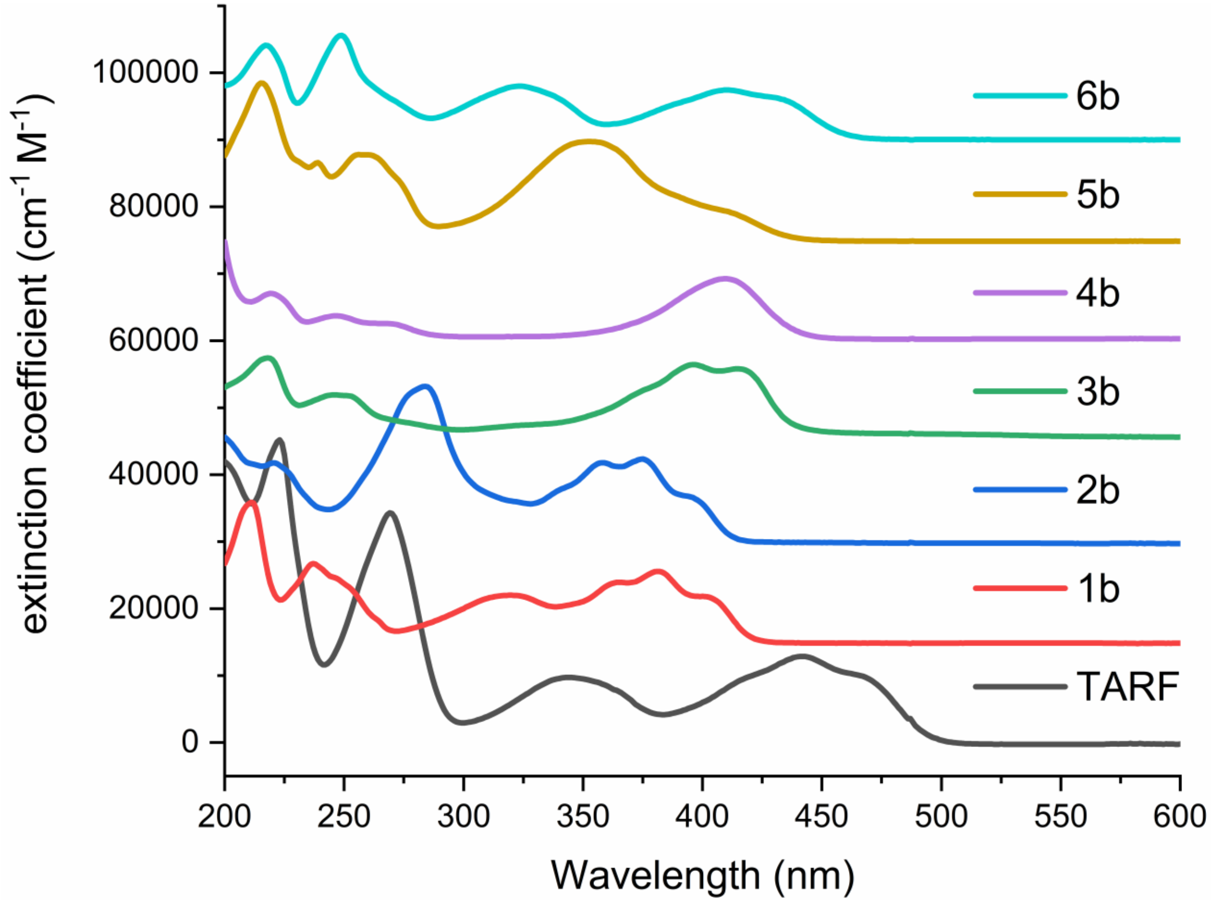

Figure S 3 UV-vis absorbance spectra of the prepared DAOF derivatives compared with TARF shown on the extinction coefficient scale (offset 15000 cm^{-1}M^{-1}). Increased electron density on DAOF leads to a bathochromic shift.

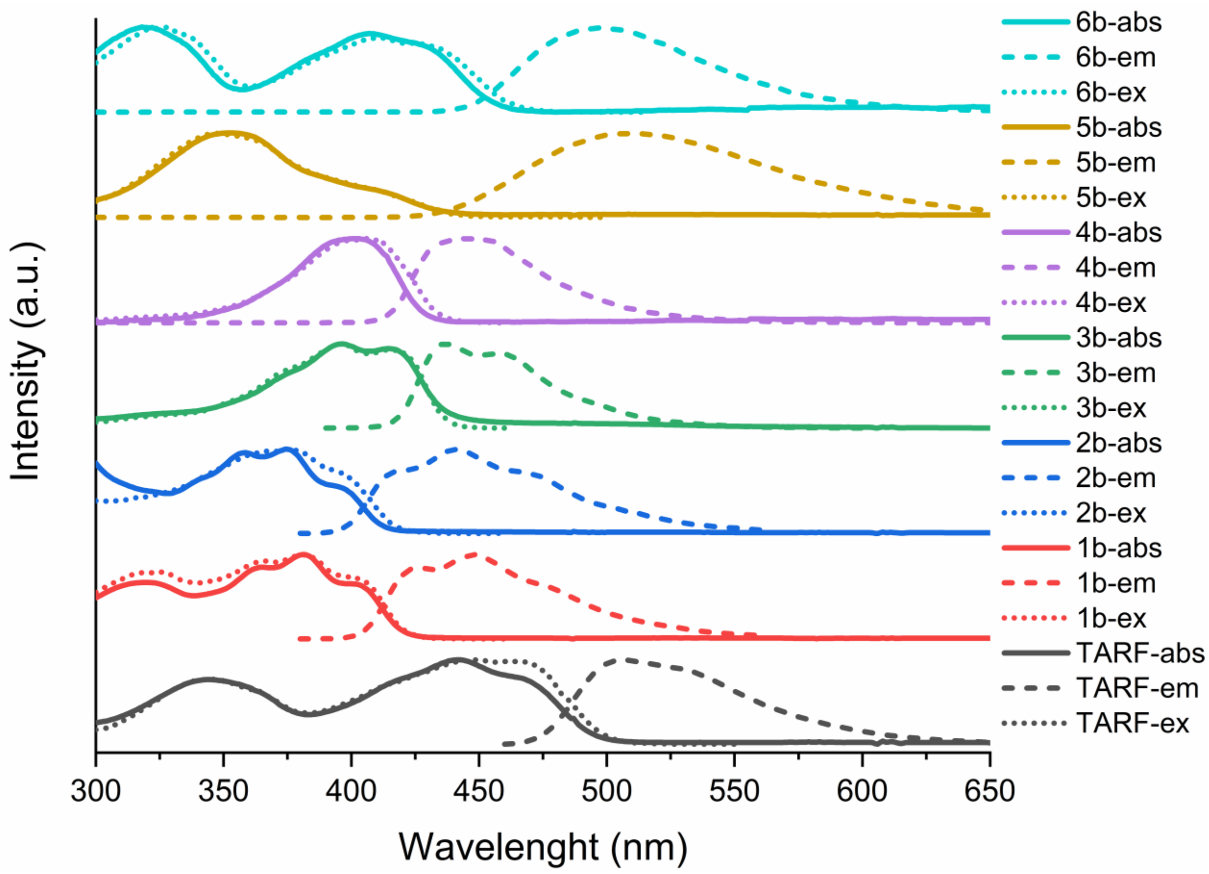

Figure S 4 Overview of absorbance, emission, and excitation spectra of DAOF derivates compared with TARF. All prepared DAOFs are hypsochromically shifted with respect to TARF. Electron donors on the DAOF skeleton lead to bathochromic shifts in both the absorbance and emission, while the nitro group has a weaker but opposite effect. abs=absorbance, em=emission, ex=excitation spectrum.

5 Photocatalysis

CAUTION: Strong LED light can cause irreversible damage to the retina. Wear appropriate eye protection. In our work, laser goggles with >OD 7 up to 532 nm were used by the involved personnel.

5.1 Catalytic oxidations

The oxidative photocatalytic properties of compounds **1-6b** were tested by oxidation of thioanisole to methyl phenyl sulfoxide. Aliquots of stock solutions of reactants in acetonitrile were added directly into the vial to achieve concentrations of compound (**b**) of 2×10^{-4} M and thioanisole of 1×10^{-2} M in a 2 mL solution of 85:15 acetonitrile:water (v:v). The vial with reagents was irradiated with a light-emitting diode at 400 nm and stirred under an oxygen atmosphere (balloon) at a stable temperature of 40 °C. The reaction course was monitored by HPLC using an Astra C18-HE 100x4.6 mm, 3 µm column and isocratic MeOH:water eluent (40:60) followed at 232 nm. The retention times for thioanisole and methyl phenyl sulfoxide were established using commercial products. Conversion was determined from peak integrals without isolation of the product. The structure of the product was confirmed by NMR analysis.

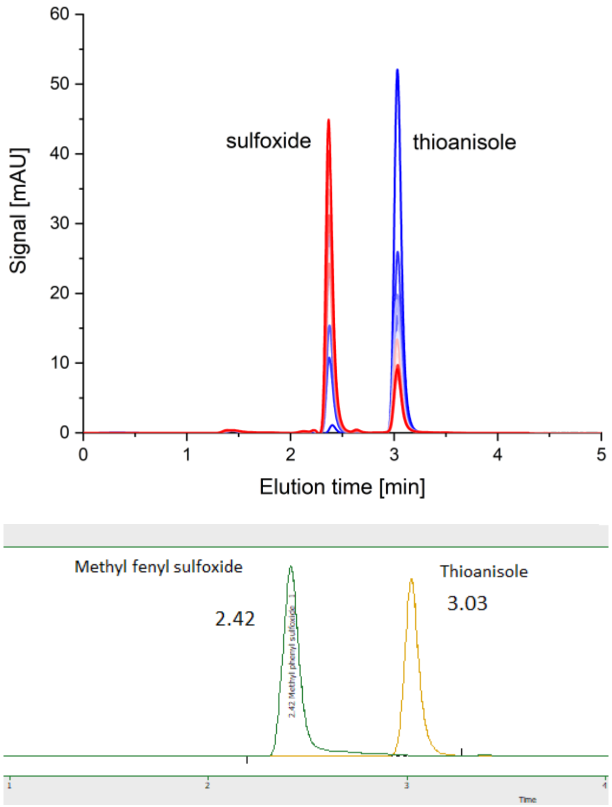

*Figure S 5 Representative chromatograms for photocatalytic oxidation using DAOF. Top: chromatograms from the photocatalytic reactions with **3b**. Bottom: overlayed chromatograms of the starting thioanisole and commercial methyl phenyl sulfoxide as the product.*

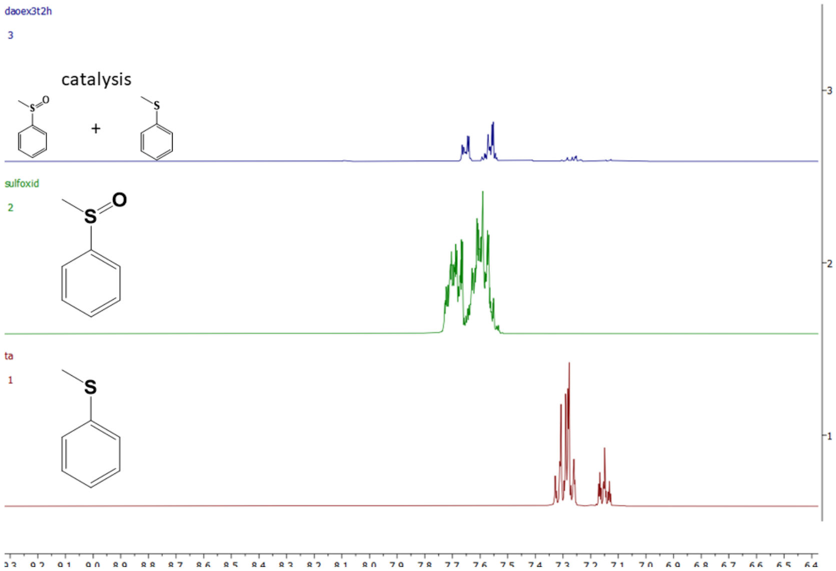

Figure S 6 NMR spectra comparing the product of photocatalytic oxidation (top trace) with the commercial reference methyl phenyl sulfoxide (middle) and the starting thioanisole (bottom trace).

Photosulfoxidation in pure acetonitrile solvent under otherwise identical conditions was performed and followed by HPLC. The reaction mixture contained the DAOF photocatalyst (**b**) at 2×10^{-4} M and thioanisole at 1×10^{-2} M. The vial with reagents was saturated with oxygen and irradiated with a 400 nm LED (temperature-stabilized at 40 °C) with stirring under an oxygen atmosphere.

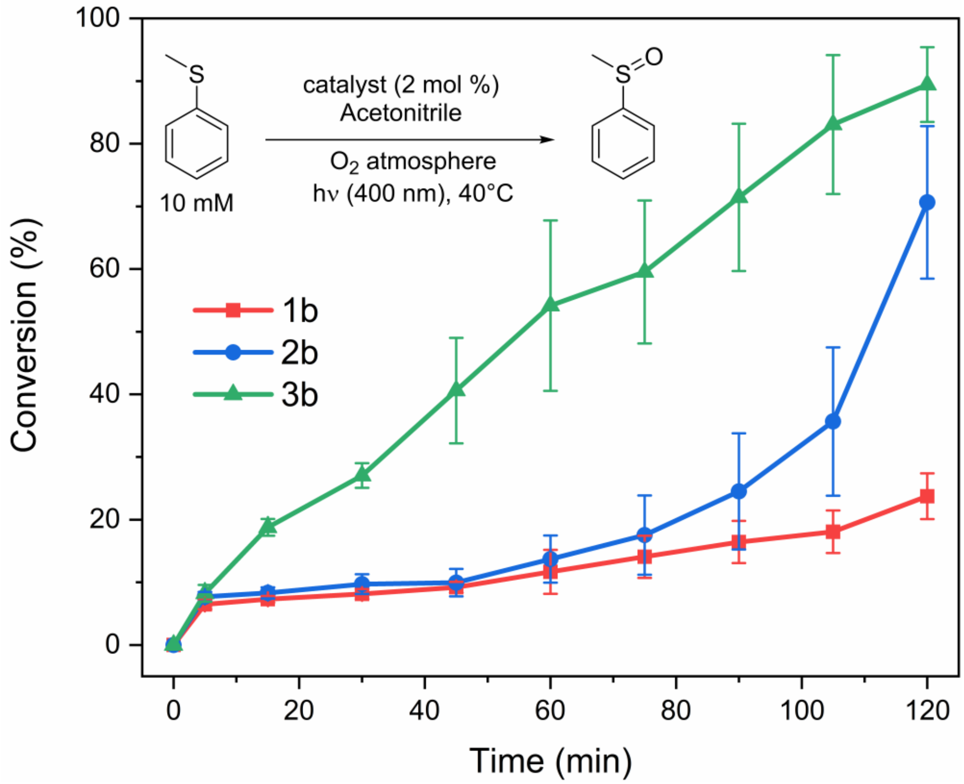

Figure S 7 Comparison of the progress of thioanisole oxidation by the prepared DAOF derivatives in pure acetonitrile as the solvent. In the inset, a scheme of the photooxidative reaction tested with the detailed conditions.

Photosulfoxidation of thioanisole using TARF as catalyst in an 85:15 acetonitrile-water mixture was performed and followed by HPLC. The conditions were set identical to the DAOF catalysts. The reaction mixture contained the TARF photocatalyst at 2×10^{-4} M and thioanisole at 1×10^{-2} M. The vial with reagents was saturated with oxygen and irradiated with a 400 nm LED (temperature-stabilized at 40 °C) with stirring under an oxygen atmosphere.

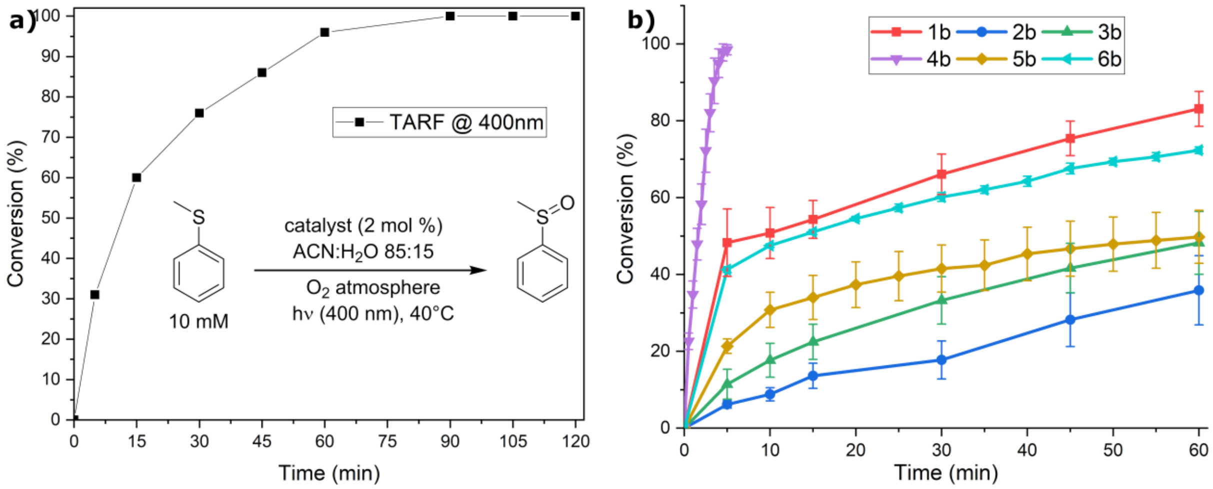

Figure S 8 a) Comparison of the progress of thioanisole oxidation by the commercial reference TARF in the same conditions as the studied catalysts. Note that 400nm is not optimal for TARF catalyst as it is usually reported in literature to work at 450nm. b) Photocatalytic oxidation of thioanisole to sulfoxide by DAOF derivatives, reproduced from Figure 3 a)

5.1.1 *Negative controls*

Negative controls were performed by omission of one component of the reaction mixture at the time. In the runs with **1-6b** when light, oxygen or catalyst was omitted, oxidation did not take place. The HPLC chromatogram did not indicate any sulfoxide peak. In the runs aiming at autonomous catalyst formation, when either light, oxygen, barbituric acid, or salicylic aldehyde was omitted, the oxidation did not take place. The HPLC chromatogram did not indicate any sulfoxide peak. Conversion data for these negative controls are reported in Figure S 9.

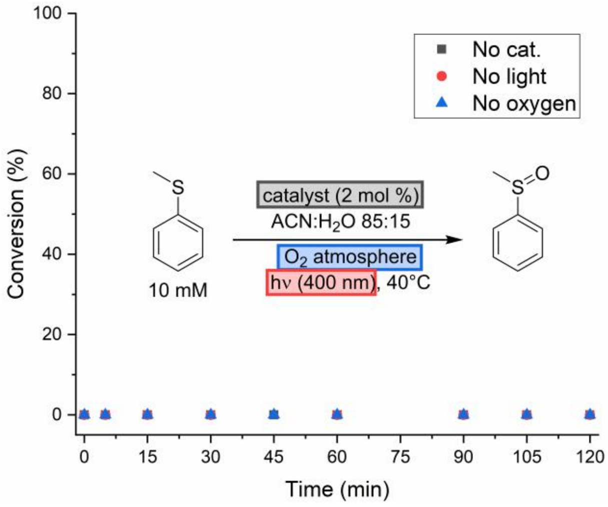

Figure S 9 Conversion points of the negative controls: black square without catalyst, red circles without light, blue triangles without oxygen. In all the cases no product of the oxidation is detected via HPLC.

5.2 *Catalytic reductions*

The reductive photocatalytic properties of compound **1b** were tested by reduction of 4-bromoanisole to anisole followed by HPLC without isolation of the products. Conversion was determined from integrated HPLC chromatograms. Retention times of starting materials and products were confirmed using commercial standards. To perform the photoreduction, a suspension containing 1 mL acetonitrile, 7.5 mM 4-bromoanisole, 2 eq. DIPEA, 1 eq. Cs_2CO_3 and 8 mol % of **1b** were prepared. By the freeze-pump-thaw method, anaerobic conditions were achieved. The vial with reagents was irradiated with a 400 nm LED (temperature-stabilized at 40 °C) and stirred. The reaction course was monitored by HPLC.

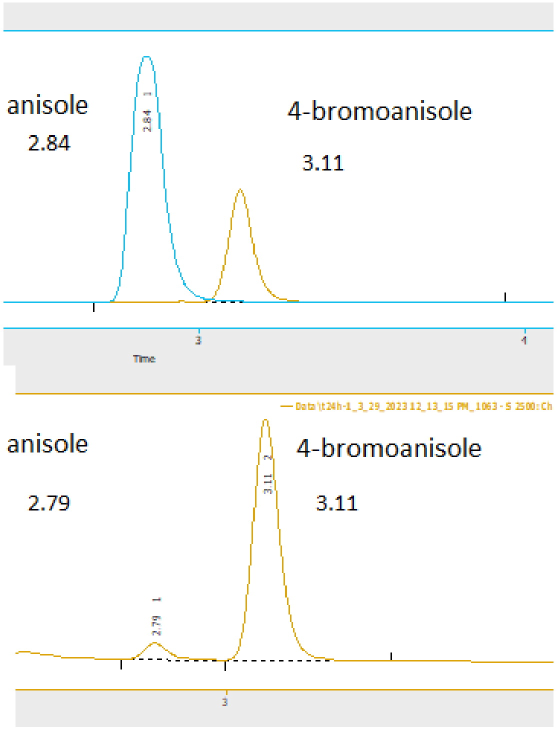

*Figure S 10 Representative chromatograms for photocatalytic reductions using DAOF. Top: overlayed chromatograms of the starting 4-bromoanisole and anisole as the product. Bottom: chromatogram from the photocatalytic reactions with **1b**.*

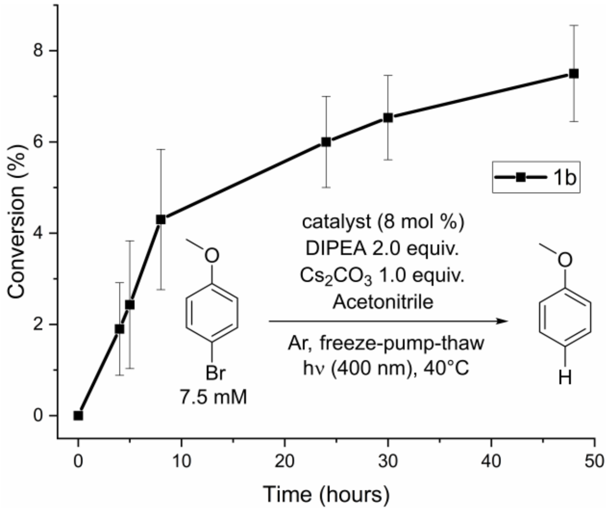

Figure S 11 Conversion of the photocatalytic dehalogenative reduction of 4-bromoanisole to anisole, average and standard deviation of three independent runs. In the inset, a scheme of the photoreductive reaction tested with the detailed conditions.

5.2.1 *Negative controls*

When either light, catalyst or DIPEA was omitted from the reaction mixture, the reduction did not take place. The HPLC chromatogram did not detect any anisole peak.

5.3 *Autonomous catalyst formation under catalytic conditions*

In a 4 mL vial, 2 mL of a solution in acetonitrile:water (85:15) containing 10 mM thioanisole, 0.2 mM salicylaldehyde and 0.2 mM barbituric acid was prepared. The mixture was saturated by oxygen by bubbling pure O_2 for 60 seconds via a syringe from a balloon. Then, the syringe was retracted above the solution surface while still under the septum of the reaction vial. Irradiation with LED while stirring was then started, and the reaction progress was monitored by HPLC. For the analysis, we withdrew 30 μL aliquots from the ongoing photocatalytic run using a Hamilton syringe and diluted them in pure HPLC grade acetonitrile to 1.5 mL, and this solution was then injected into the HPLC system.

Spontaneous formation of **1a** and **1b** from barbituric acid and salicylaldehyde was confirmed by high-resolution mass spectrometry. HRMS spectra (Figure S 12 and Figure S 13) are presented in relative intensities *vs* the strongest peak. The presence of crucial species **1a** and **1b** was unequivocally confirmed.

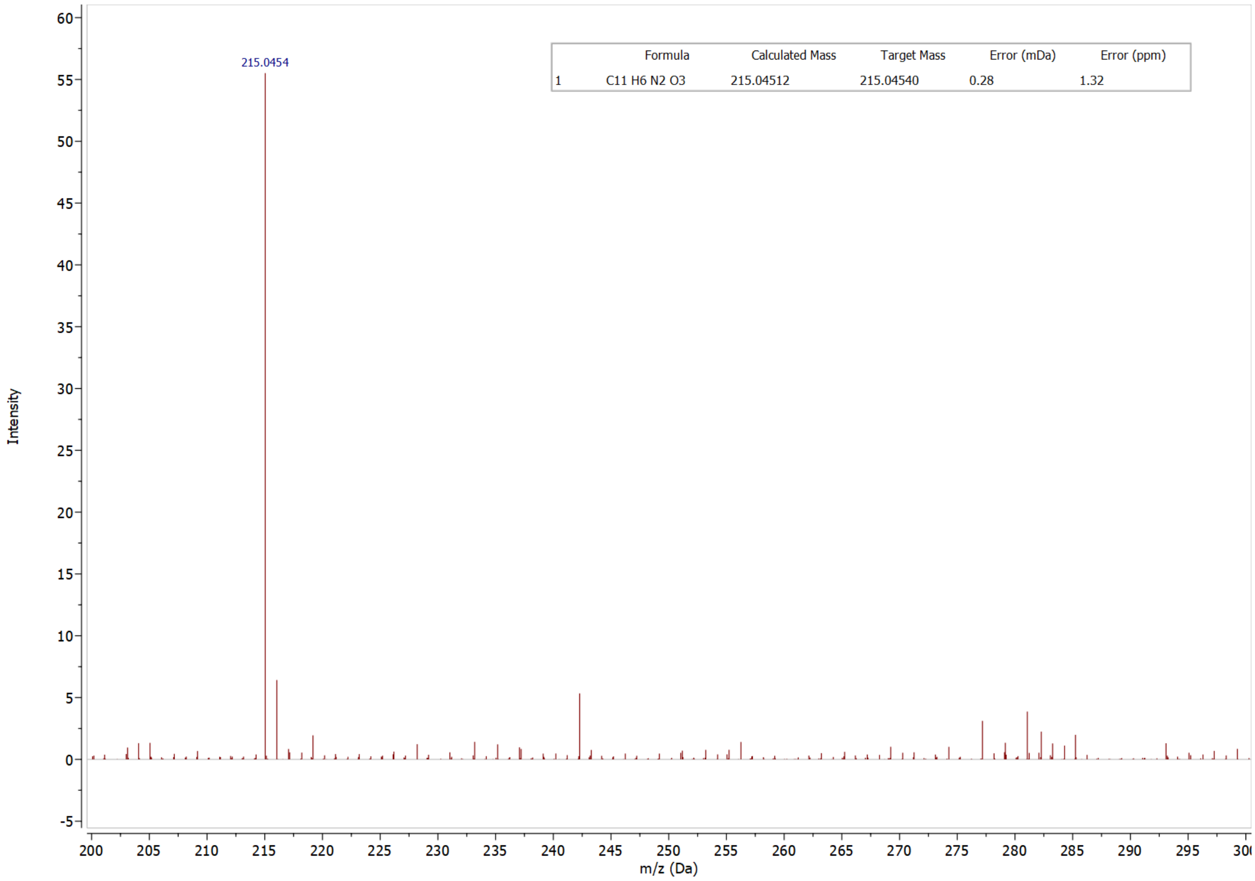

*Figure S 12 HRMS spectrum with APCI⁺ ionization of the reaction mixture containing salicylaldehyde, barbituric acid, thioanisole and oxygen after irradiation with 400 nm light for 3 hours. The mass of **1b** is identified as [M+H]⁺ within 1.3 ppm error, which unambiguously shows the presence of **1b** in the mixture.*

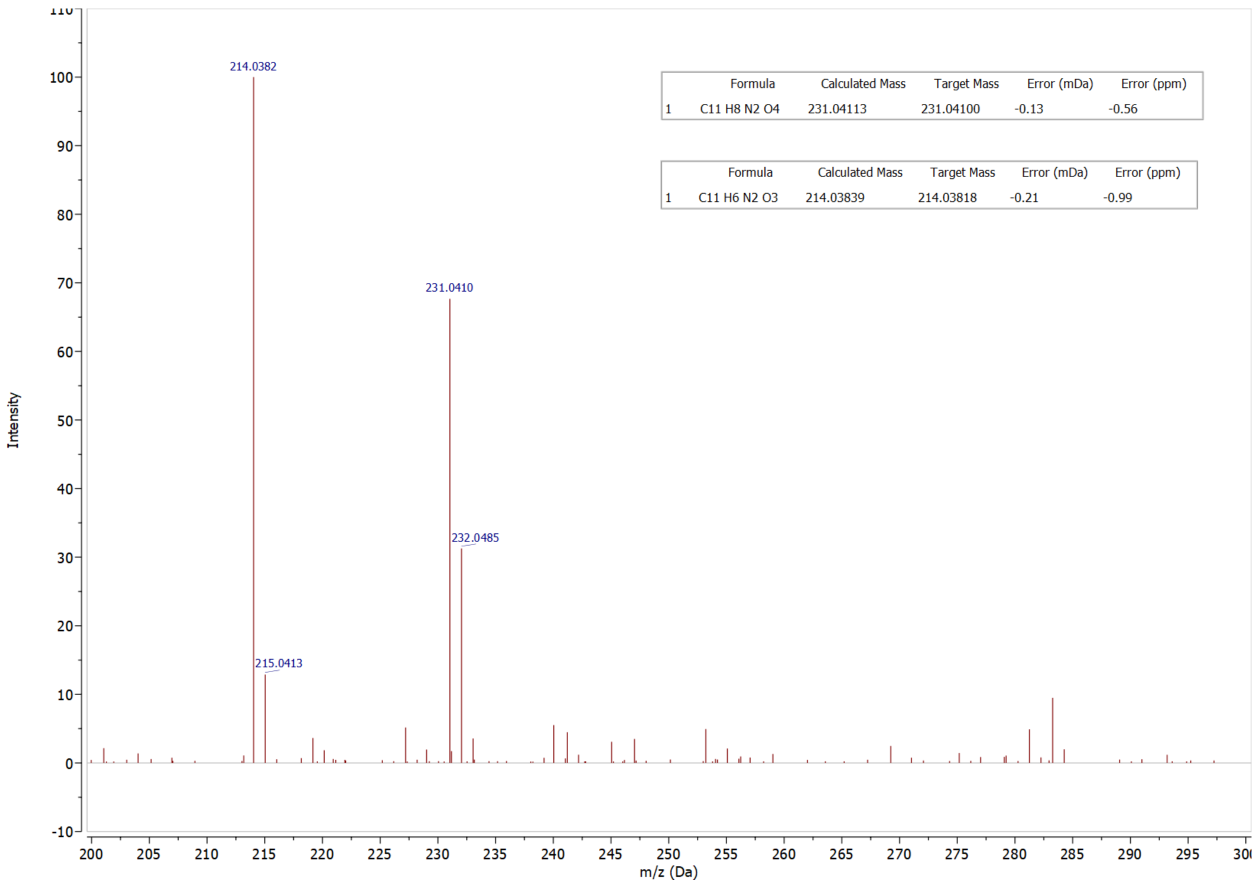

*Figure S 13 HRMS spectrum with APCI ionization of the reaction mixture containing salicylaldehyde, barbituric acid, thioanisole and oxygen after irradiation with 400 nm light for 3 hours. The mass of **1a** is identified as [M-H]$^-$ within 1 ppm error, which unambiguously shows the presence of **1a** in the mixture. **1b** ionizes well as an M$^-$ ion, showing its capacity to accommodate an electron within its structure.*

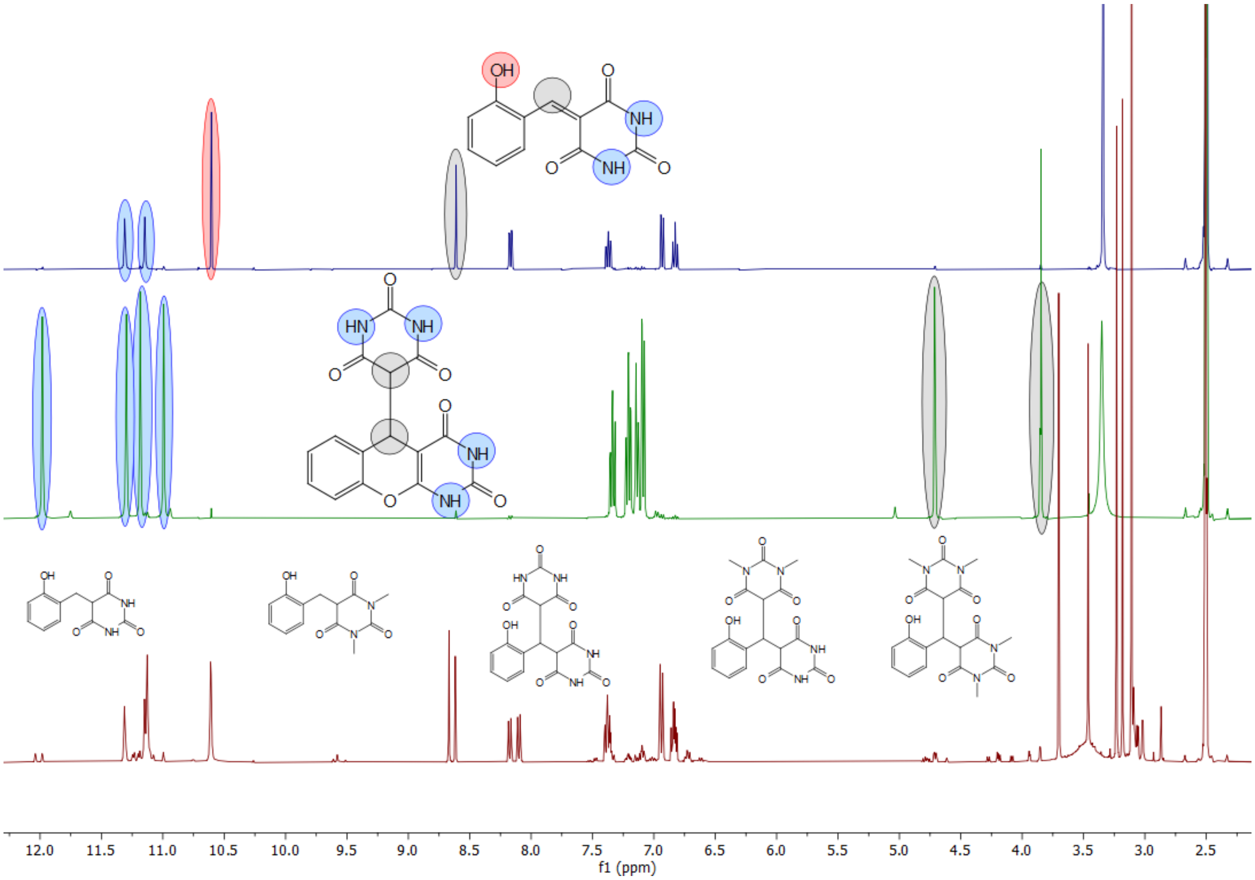

Figure S 14 Stacked NMR spectra showing the covalent dynamicity of DAOF derivatives. Top blue trace shows NMR spectrum of ***1a*** *in DMSO-d6. The bottom red trace shows the NMR spectrum of a DMSO-d6 solution of* ***1a*** *(20 mM), to which 1 eq. of N,N'-dimethylbarbituric acid was added. Equilibrium was established over a period of 48 hours at ambient temperature and consisted of 37 %* ***1a****, 35 % its N,N'-dimethylated analogue and three adducts of aldehyde with two barbiturates (nonmethylated 10 %, dimethylated 7 % and tetramethylated 11 %).* ***1b*** *also features the Michael acceptor, and indeed, the addition of a second barbituric moiety proceeds in DMSO-d6 with full conversion within a minute, so equilibrium is reached before the first NMR spectrum can be acquired (green trace).*

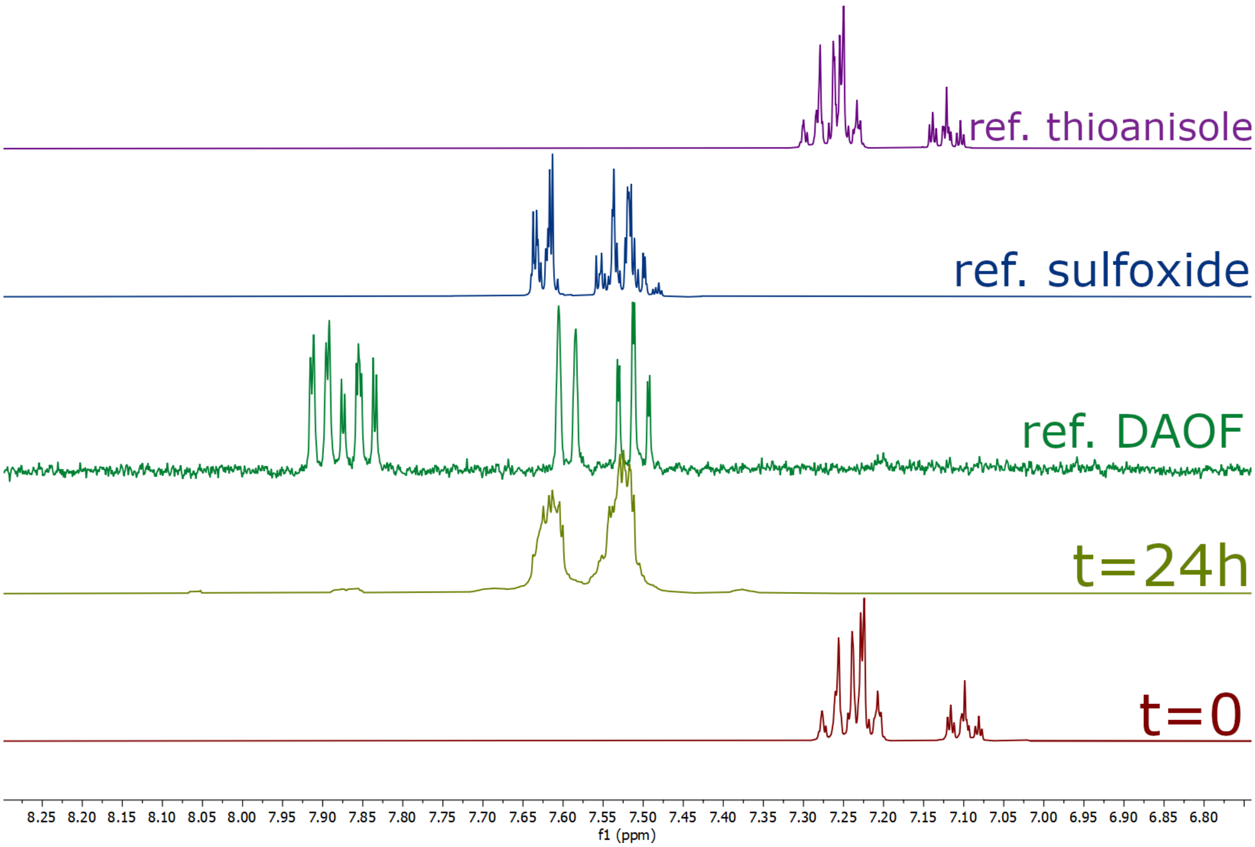

Figure S 15 NMR spectra of the mixture containing 0.2 mM salicylaldehyde and barbituric acid and 10 mM thioanisole in CD_3CN/D_2O (85:15 v/v) right after mixing (bottom brown line) and after 24 hours of irradiation by 400 nm LED (light green line). Reference NMR spectra of DAOF ***1a****, starting thioanisole and the photosulfoxidation product are given for comparison. Complete conversion of thioanisole is observed. Weak signals at around 7.9 ppm confirm the formation of DAOF derivatives.*

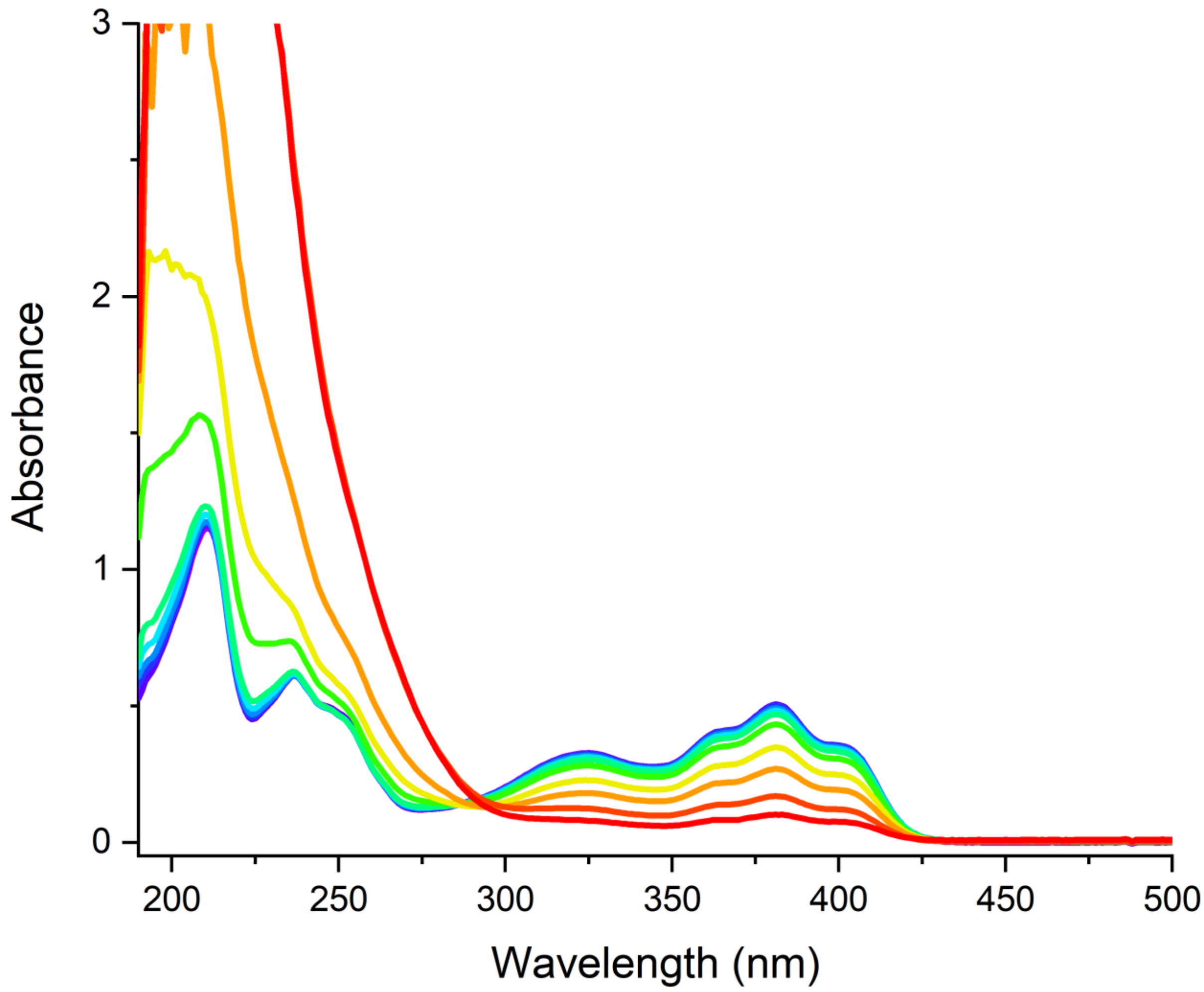

Figure S 16 UV-vis spectra of ***1b*** *in the mixture of acetonitrile-water (85:15 v/v) after addition of hydrogen peroxide. Traces are color-coded in gradient from blue (start) to red (end). Gradual decrease of the most bathochromically shifted absorbance feature (ca 300-420 nm) is observed in agreement with decrease of conjugated system, as expected for Michael addition of* H_2O_2 *on* ***1b****.*

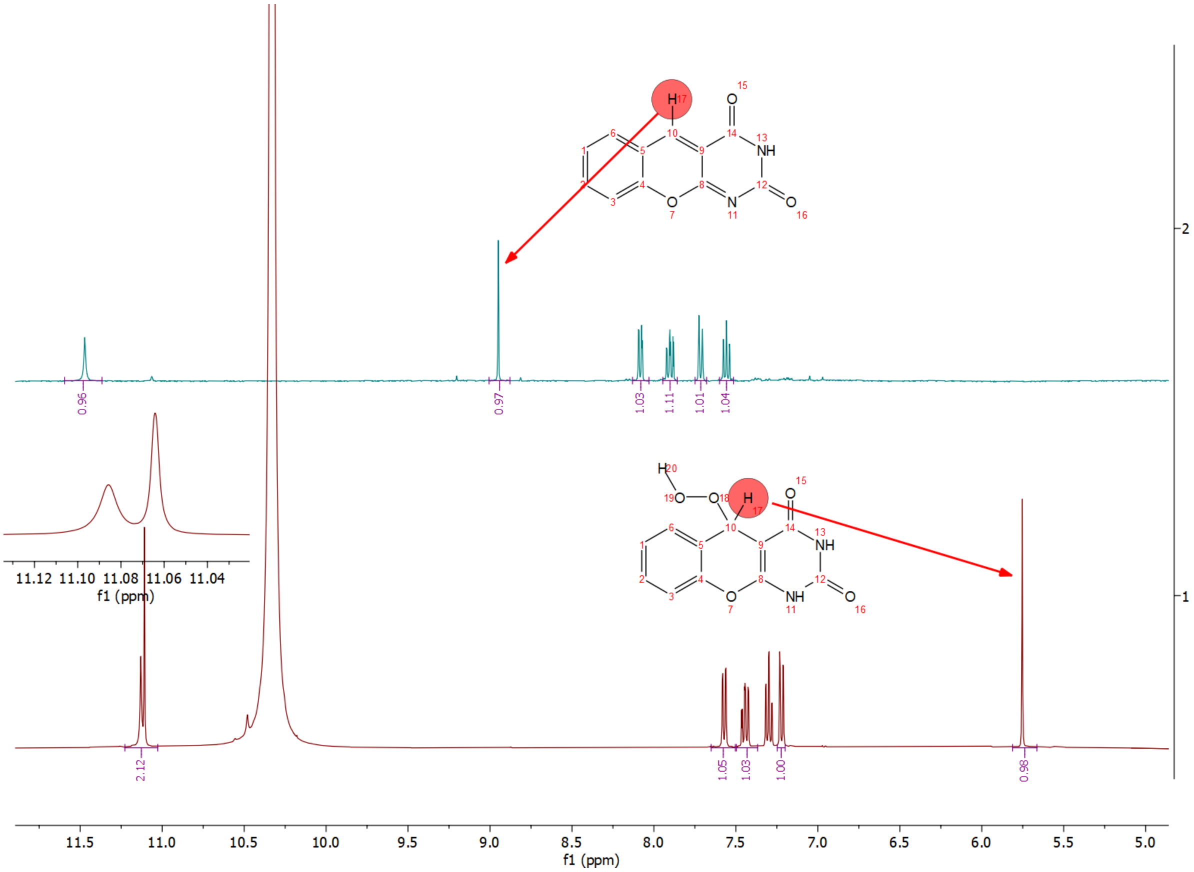

*Figure S 17 NMR spectra of **1b** in d6-DMSO (top trace) and **1b** after addition of aqueous H_2O_2 (bottom trace). The signal of the hydrogen atom H17 has a shift of 8.95 ppm, but it changes its character to benzylic after addition of the hydrogen peroxide and appears at 5.71 ppm, i.e. in the similar range as benzyl alcohol. The NH signal also shifts from 11.5 to 11.1 ppm and increases in intensity corresponding to two protons in the adduct with H_2O_2, as expected for the Michael addition product. Closer inspection of this peak (inset in bottom trace) shows that it contains two peaks. Their relative intensity is 1:1, however, the signal at 11.09 ppm has line width of 3.96 Hz and the one at 11.06 ppm has line width of 1.63 Hz. This indicates dynamic exchange process. While proton exchange with the solvent has equal probability for both NH protons, the reversibility of the Michael-retro-Michael adds broadening to the signal at 11.09 ppm.*
